# HMG-box transcription factor 1: a positive regulator of the G1/S transition through the Cyclin-CDK-CDKI molecular network in nasopharyngeal carcinoma

**DOI:** 10.1038/s41419-017-0175-4

**Published:** 2018-01-24

**Authors:** Shiwei He, Sheng Yang, Man Niu, Yancheng Zhong, Dan Gao, Yanru Zhang, Haotian Ma, Wei Xiong, Ming Zhou, Yanhong Zhou, Bo Xiang, Guiyuan Li, Cijun Shuai, Shuping Peng

**Affiliations:** 1 Hunan Provincial Tumor Hospital and Tumor Hospital of Xiangya School of Medicine, Xiangya Hospital, Central South University, Changsha, Hunan China; 2 The Key Laboratory of Carcinogenesis and Cancer Invasion of the Chinese Ministry of Education, Cancer Research Institute, Central South University, Changsha, Hunan China; 3 Human Reproduction Center, Shenzhen Hospital of Hongkong University, Shenzhen, Guangdong China; 4 StateKey Laboratory of High Performance Complex Manufacturing, College of Mechanical and Electrical Engineering, Central South University, Chang Sha, Hunan China

## Abstract

HMG-box transcription factor 1 (HBP1) has been reported to be a tumor suppressor in diverse malignant carcinomas. However, our findings provide a conclusion that HBP1 plays a novel role in facilitating nasopharyngeal carcinoma (NPC) growth. The Kaplan–Meier analysis indicates that high expression HBP1 and low miR-29c expression both are negatively correlated with the overall survival rates of NPC patients. HBP1 knockdown inhibits cellular proliferation and growth, and arrested cells in G1 phase rather than affected cell apoptosis via flow cytometry (FCM) analysis. Mechanistically, HBP1 induces the expression of CCND1 and CCND3 levels by binding to their promoters, and binds to CDK4, CDK6 and p16^INK4A^ promoters while not affects their expression levels. CCND1 and CCND3 promote CCND1-CDK4, CCND3-CDK6, and CDK2-CCNE1 complex formation, thus, E2F-1 and DP-1 are activated to accelerate the G1/S transition in the cell cycle. MiR-29c is down-regulated and correlated with NPC tumorigenesis and progression. Luciferase assays confirms that miR-29c binds to the 3′ untranslated region (3′-UTR) of HBP1. Introduction of pre-miR-29c decreased HBP1 mRNA and protein levels. Therefore, the high endogenous HBP1 expression might be attributed to the low levels of endogenous miR-29c in NPC. In addition, HBP1 knockdown and miR-29c agomir administration both decrease xenograft growth in nude mice in vivo. It is firstly reported that HBP1 knockdown inhibited the proliferation and metastasis of NPC, which indicates that HBP1 functions as a non-tumor suppressor gene in NPC. This study provides a novel potential target for the prevention of and therapies for NPC.

## Introduction

Nasopharyngeal carcinoma (NPC) is the most common cancer originating in the nasopharynx and predominant in Southeast Asia and Africa, especially in South China^1,2^. From the statistical data on cancers in 2015, ~60,000 new cases were diagnosed and 34,000 patients with NPC died in China. Almost 22% of all new NPC cases in the world and 27% of deaths from NPC are in China^3,4^. Susceptibility to NPC is complicated, includes genetic modifications (racial predisposition, family aggregation, and geographical concentration), viral infection (Epstein-Barr virus, EBV) and environmental factors^5–8^.

MiR-29c is a member of the miR-29 family, which inhibits NPC invasion and metastasis in several studies^9,10^. We also found that miR-29c regulates the miR-34c and miR-449 expression by targeting DNMT3a and DNMT3b in NPC cells^10^. HBP1 (HMG-box transcription factor 1) is another tentative target gene of miR-29c. HBP1 is a transcription factor that contains a HMG-box (DNA-binding domain). It was firstly cloned from rat brains, and its functions were initially confirmed in cell differentiation and premature senescence^11–13^. HBP1 regulates the timing of neuronal differentiation through downstream genes such as cyclin D1 (CCND1), a downstream signal molecule in the Wnt signaling pathway. HBP1 also plays important roles in the development and progression of malignant diseases^14–16^. Chen, Y. et al. reported that HBP1 enhances the radiation sensitivity of prostate cancer cell by promoting cells apoptosis during radiation treatment^17^. HBP1 inhibits the Wnt/β-catenin signaling pathway by inhibiting the activity of LEF/TCFs and preventing β-catenin from being transported into the nucleus and inhibits the growth of HCT116 and Caco-2A colon cancer cells^18–21^. However, the role of HBP1 in NPC has not been defined yet.

In this study, we accidently found that HBP1 is highly expressed in NPC cell lines and tissues, and negatively correlated with NPC patient’s survival time. HBP1 knockdown inhibited the growth, proliferation, invasion and metastasis of NPC cells in vitro. We further confirmed that HBP1 acted as a target gene of miR-29c. We also demonstrated that HBP1 was recruited to the CCND1, CCND3, CDK4, CDK6, and p16 promoters. HBP1 knockdown reduced CCND1 and CCND4 expression levels and increased the expression p21 and p27 expression levels in NPC cells. HBP1 knockdown and the miR-29c agomir treatment both attenuated the growth and metastasis of NPC xenografts in nude mice in vivo. This is the first report to show that HBP1 may have a novel tumor-promoting role in NPC development and invasion.

## Results

### HBP1 is highly expressed in NPC tissues or cell lines

**Fig. 1:**
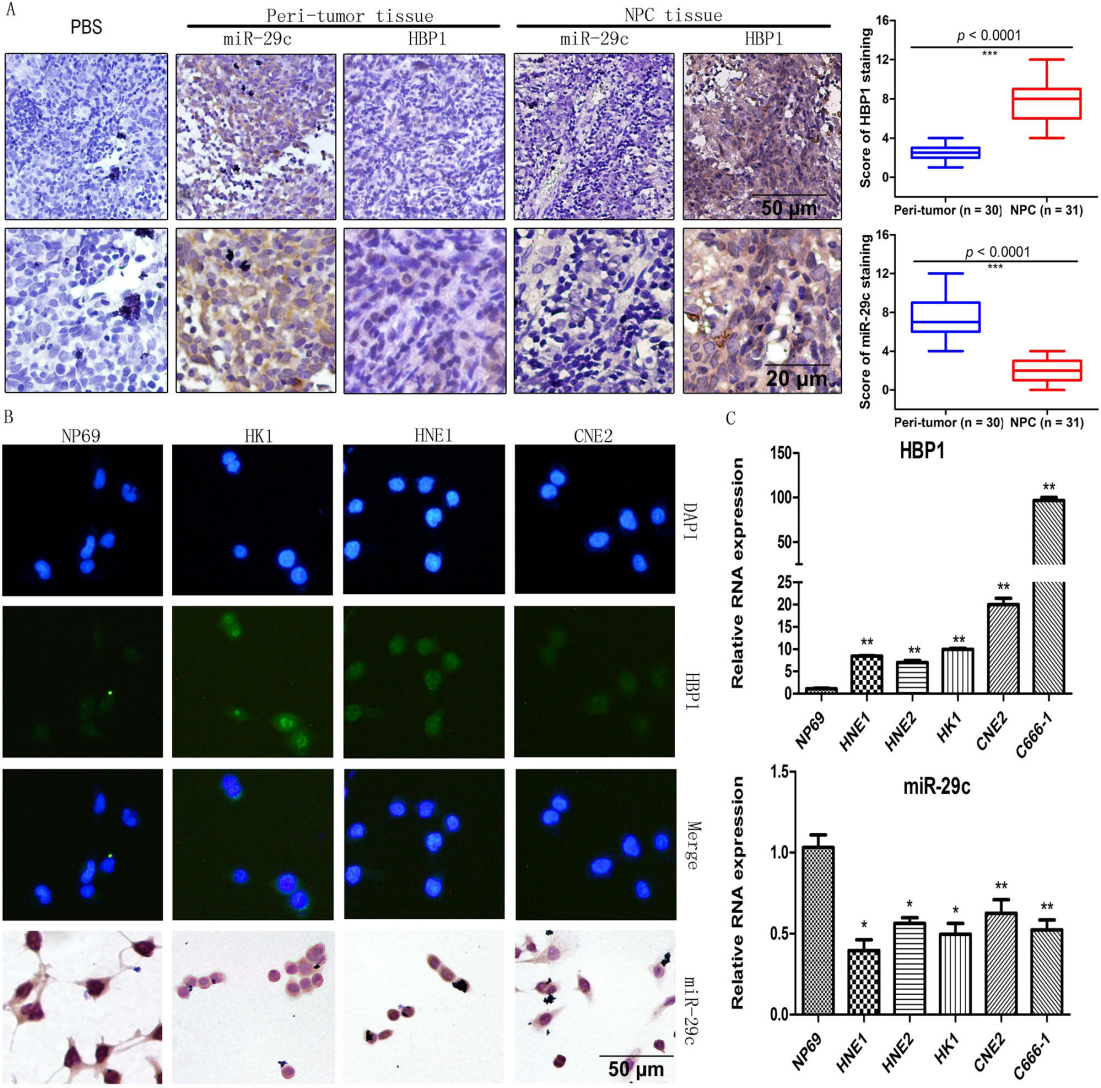
Dysregulated expression of HBP1 and miR-29c in NPC tissues or cell lines **a** In situ hybrization and immunohistochemistry assays analysis for miR-29c and HBP1 expression in NPC tissues. Scale bars: upper, 50 μm; lower, 20 μm. Right: Statistical analysis for HBP1 and miR-29c expression between peri-tumor and NPC tissues. HBP1, *p < *0.0001, miR-29c, *p* < 0.0001. **b** Immunofluorescence analysis for HBP1 (green) and in situ hybrization analysis for miR-29c (brown) in NP69, HK1, HNE1, and CNE2 cells. Scale bars, 50 μm. **c** RT-qPCR analysis for HBP1 and miR-29c levels in NP69, HNE1, HNE2, HK1, CNE2, and C666–1 cell lines. GAPDH was used as an internal control for mRNA and U6 was used as an internal control for miRNA

It has been reported that miR-29c is a suppressor and expressed at a very low levels in various tumors and down-regulated in NPC cell lines^10^. We found that miR-29c had low expression in NPC tissues compared with peri-tumor tissues (Fig. 1a). However, it was very surprising and unexpected to find that HBP1 was up-regulated in NPC tissues (Fig. 1a). Among 31 NPC tissues, 21 NPC tissues is high HBP1 expression and 10 NPC tissues is low HBP1 expression, which show a HBP1 high expression rate (67.74%) in NPC. Immunofluorescence results suggested that HBP1 exhibited higher fluorescence intensity in these HK1, HNE1, and CNE2 cells than in normal nasopharyngeal epithelial cells (NP69) and mainly located in the cell nucleus, while in situ hybridization results revealed that miR-29c was distributed in both the cytoplasm and nucleus at low miRNA levels (Fig. 1b). HBP1 mRNA levels were significantly higher in NPC cell lines (HNE1, HNE2, CNE2, C666–1, and HK1) than in NP69 cells, specifically, the HBP1 mRNA levels changed by 20-fold in CNE2 cells and by 100-fold in C666–1 cells (Fig. 1c). In contrast, miR-29c miRNA levels were lower in NPC cell lines than in NP69 cells (Fig. 1c). We also have detected HBP1 protein expression levels in NPC and non-NPC cells by western blotting. The results showed that HBP1 is highly expressed in NPC cells, while lowly expressed in liver cancer cells QYG7703, SMMC7721, and HepG2 compared to the human hepatocyte line QSG7701, and breast cancer cells MCF-7, T47D and MDA-MB-231 compared to the human mammary epithelial cell line MCF-10A, and ovarian cancer cells, A2780 and PA-1 compared to the human ovarian epithelial cell line IOSE-80 (Fig. S1f).

### HBP1 expression is positively correlated with advanced clinicopathological implications in NPC patients

**Fig. 2:**
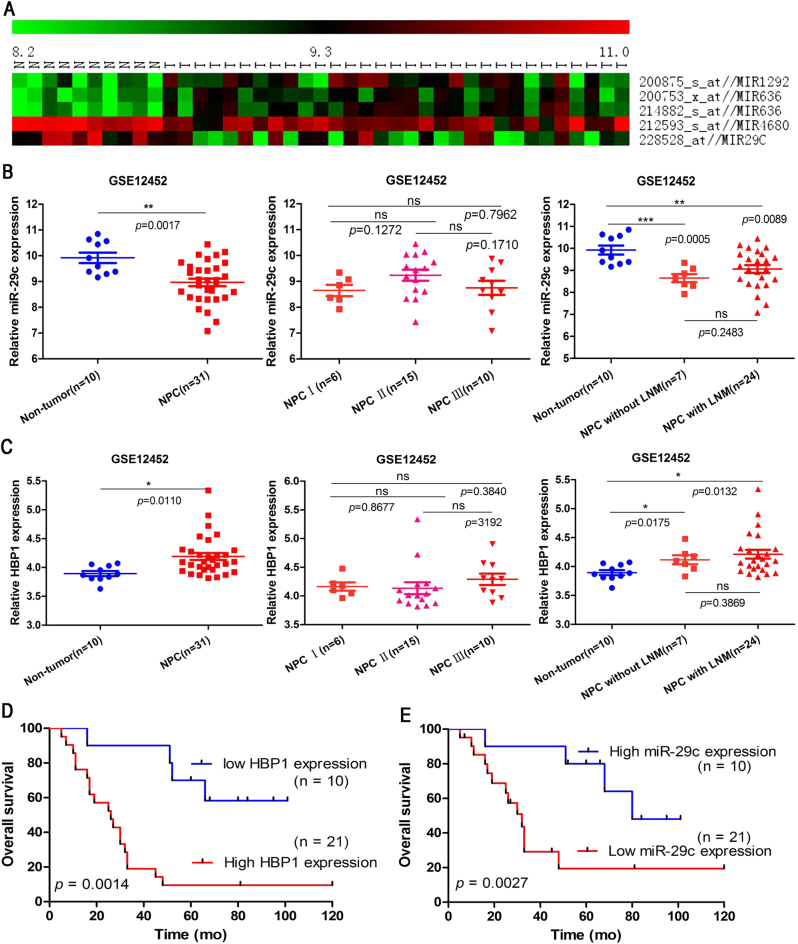
HBP1 is correlated with clinicopathological implications on patients with NPC **a** Heat map of 5 dys-regulated miRNAs were filtered from the GSE12452 data set. **b** MiR-29c is down-regulated in NPC biopsies (*n* = 31) compared with non-tumor NPE tissues (*n* = 10) in the GSE12452 dataset. MiR-29c expression levels were not associated with tumor-nodes-metastasis (TNM) stage (I, *n* = 6; II, *n* = 15, III, *n* = 15) and lymph node metastasis (LNM) (non-tumor, *n* = 10; NPC without LNM, *n* = 7; NPC with LNM, *n* = 24). **c** HBP1 is up-regulated in NPC biopsies (*n* = 31) compared with non-tumor NPE tissues (*n* = 10) in the GSE12452 dataset. HBP1 expression levels were not associated with TNM stage (I, *n* = 6; II, *n* = 15, III, *n* = 15) and LNM (non-tumor, *n* = 10; NPC without LNM, *n* = 7; NPC with LNM, *n* = 24). **d** Overall survival rates of 31 NPC patients were compared between the low HBP1 and high HBP1 expression groups, *p* = 0.0012. **e** Overall survival rates of 31 NPC patients were compared between the low miR-29c and high miR-29c expression groups, *p* = 0.0027

To clarify whether HBP1 is relevant to clinicopathological implications in patients with NPC, we first conducted bioinformatics analysis using a NPC gene microarray data from the Affymetrix Human Genome U133 Plus 2.0 Array platform (HG-U133_Plus_2) in GEO Datasets (GEO accession: GSE12452). Five miRNAs were found to be remarkably dysregulated expression among 48 miRNAs from GSE12452 (Table S1 and S2). MiR-1292 and miR-636 were up-regulated, while miR-4680 and miR-29c were down-regulated (Fig. 2a). Moreover, we found that miR-29c had lower expression in NPC tissues than in NPE tissues (Fig. 2b, the left side), while HBP1 had higher expression in NPC tissues than in NPE tissues (Fig. 2c, the left side). However, miR-29c and HBP1 gene expression in NPC patients was not significantly different between the different tumor TNM stages, and NPC patients with and without lymphatic metastasis (LNM) (Fig. 2b, c, both the middle and right side). Next, we have performed bioinformatics analysis for our collected NPC specimens, which show that NPC patients with high HBP1 expression have a higher TNM stage (TNM III-IV) than those with low HBP1 expression (TNM I-II) (Table 1). Cox analysis also shows that the mortality of NPC patients with high HBP1 expression is more than 2.5 times that of those with low HBP1 expression (Table 2). Furthermore, Kaplan–Meier survival analysis suggests that high HBP1 expression is negatively correlated with the overall survival rates of NPC patients, while high miR-29c expression is positively correlated with the overall survival rates of NPC patients (Fig. 2d, e).

**Table 1:** Relationship between HBP1 expression and clinicopathologic features of NPC patients (*n* = 31)

Variable	HBP1		
	Low expression (N)	High expression (N)	*p* value
Sex			
Male	8	13	0.0698
Female	2	8	
Age (yr)			
≤45	3	9	0.1968
≥46	7	12	
TNM Stage			
TNM I-II	6	7	0.0375*
TNM III-IV	4	14	
Primary therapy			
Radio therapy	8	13	0.0536
Chemotherapty	2	8	
Radio-chemtherapy	10	19	

*TNM* tumor-nodes-metastasis *p* Values for clinical variables were assessed by *χ*^2^ test

*Bold values indicate statistically significant values

**Table 2:** Cox analysis of clinical variables contributing to overall survival

Analysis	Variable	B	SE	Waid	Df	Sig.	Exp(B)	95% Cl in Exp(B)	
								Down	Up
Step1	Age	.009	.016	.313	1	.576	1.009	.977	1.042
	Sex	.108	.406	.070	1	.791	1.114	.502	2.470
	Histopathological typing	–.274	.592	.215	1	.643	.760	.238	2.425
	TNM stage	.645	.488	1.751	1	.186	1.907	.733	4.961
	Therapies	–.811	.278	8.521	1	.004*	.445	.258	.766
	Lymphatic metastasis	.101	.664	.023	1	.879	1.106	.301	4.064
	HBP1	1.081	.402	7.227	1	.007*	2.947	1.340	6.480
Step2	Therapies	–.682	.250	7.447	1	.006*	.506	.310	.825
	HBP1	1.020	.387	6.944	1	.008*	2.774	1.299	5.927

*HR = Exp (B)* Hazard ratio *p* Values for clinical variables are from Cox regression after adjusting for each other

*Bold values indicate statistically significant values

### HBP1 is negatively regulated by miR-29c

**Fig. 3:**
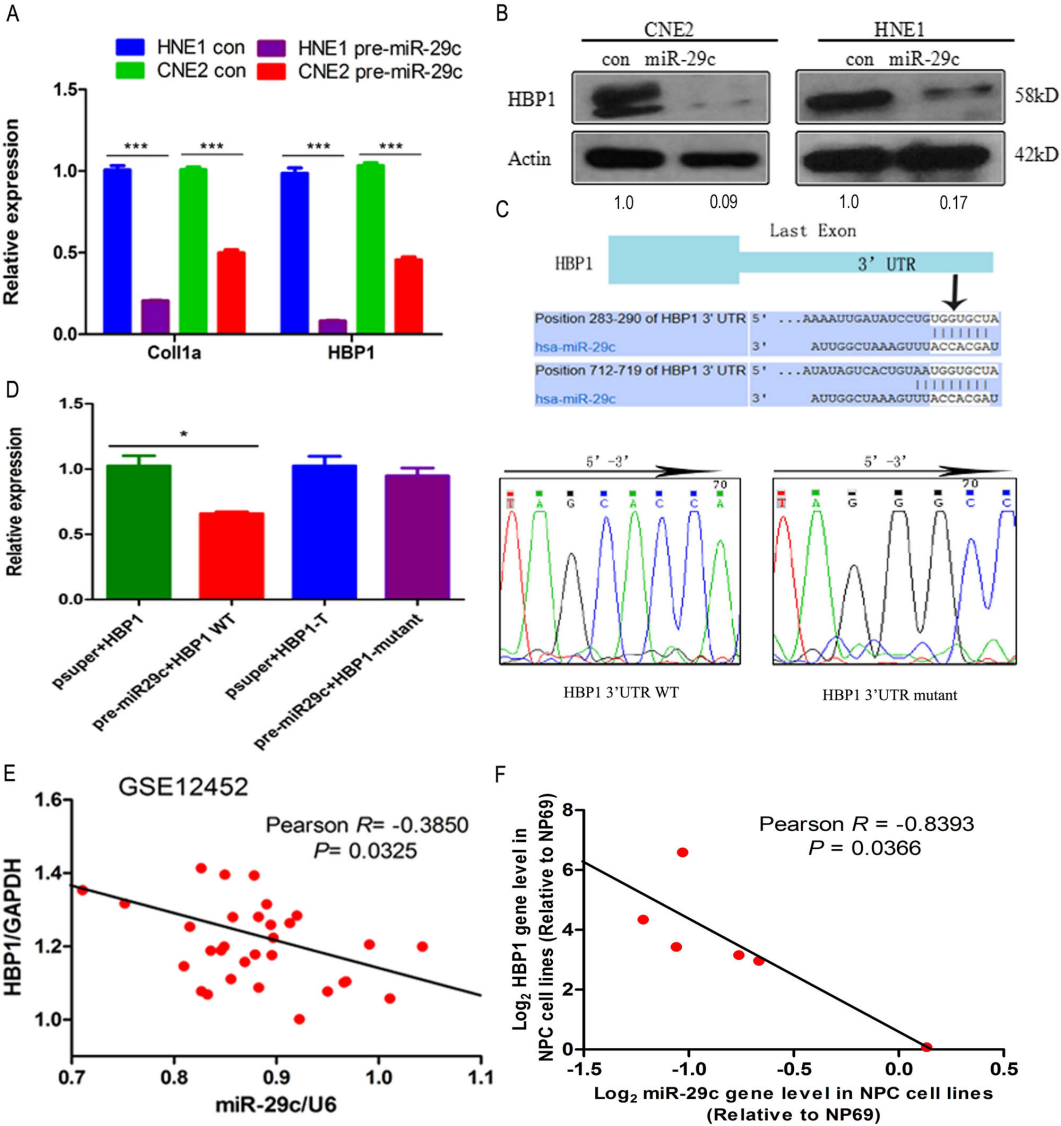
HBP1 is negatively regulated by miR-29c **a** and **b** RT-qPCR and western blotting assays for detecting HBP1 mRNA and protein expression levels in HNE1 and CNE2 cell lines after transfection with pre-miR-29c. Coll1a is regarded as a positive target of miR-29c. **c** Upper: Prediction of binding sites between hsa-miR-29c and the HBP1 3’UTR (Untranslated Region) by Target Scan system. Lower: Sequencing results of miR-29c seed sequences which binding to HBP1 3’UTR wild type (WT) and HBP1 3’UTR mutant. **d** Luciferase activity of 293T cells transfected with luciferase constructs containing HBP1 3’ UTR wild-type or mutation seed sequences of miR-29c-targeted, *p = *0.009. **e** MiR-29c is inversely correlated with HBP1 expression in NPC tissues from GSE12452 data set (Pearson *R = *−0.3850, *p* = 0.0325). **f** MiR-29c is correlated negatively with HBP1 expression in the NPC cell lines (Pearson *R = *−0.8393, *p* = 0.0366). Basal miR-29c levels were normalized to U6 and basal HBP1 levels were normalized to GAPDH in NPC cell lines (Fig. 1c), Log_2_ transformed

Due to the expression pattern between miR-29c and HBP1 was opposite in NPC, we performed luciferase assays to find out whether HBP1 is a target gene of miR-29c. HBP1, HDAC1, HDAC2, and HDAC4 were initially selected as tentative target genes by the miRanda^22^ and TargetScan^23^ miRNA prediction programs (Table S3). Ectopic expression of miR-29c by miR-29c precursor vector (pre-miR-29c) reduced HBP1, HDAC1, HDAC2, and HDAC4 mRNA expression at different levels (Fig. S1a). Transfection with pre-miR-29c caused a distinct decrease in HBP1 mRNA and proteins levels (Fig. 3a, b). Prediction programs show that MiR-29c has two identical binding sites in the HBP1 3′-UTR region, from 283 to 290 bp and 712 to 719 bp (Fig. S1b–d). Thus, we constructed firefly luciferase reporter genes which contain the wild-type (WT) or the mutant HBP1 3′-UTR sequence (Fig. 3c). The luciferase activity of the HBP1 WT 3′-UTR group were decreased, while the HBP1 mutant 3′-UTR group were no significant changed (Fig. 3d). To understand the relationship between miR-29c and HBP1 expression, we conducted correlation analysis among the NPC tissues and cell lines. From the GSE12452 data, the abundance of miR-29c was negatively correlated with HBP1 expression (Fig. 3e), and the endogenous HBP1 levels were highly negatively correlated with endogenous miR-29c levels in the cell lines panel (Fig. 3f). The high endogenous HBP1 expression might be attributed to the low levels of endogenous miR-29c in NPC (Figs. S2a–c).

### HBP1 promotes NPC cell proliferation and invasion, which can be reversed by introduction of pre-miR-29c or miR-29c mimics

**Fig. 4:**
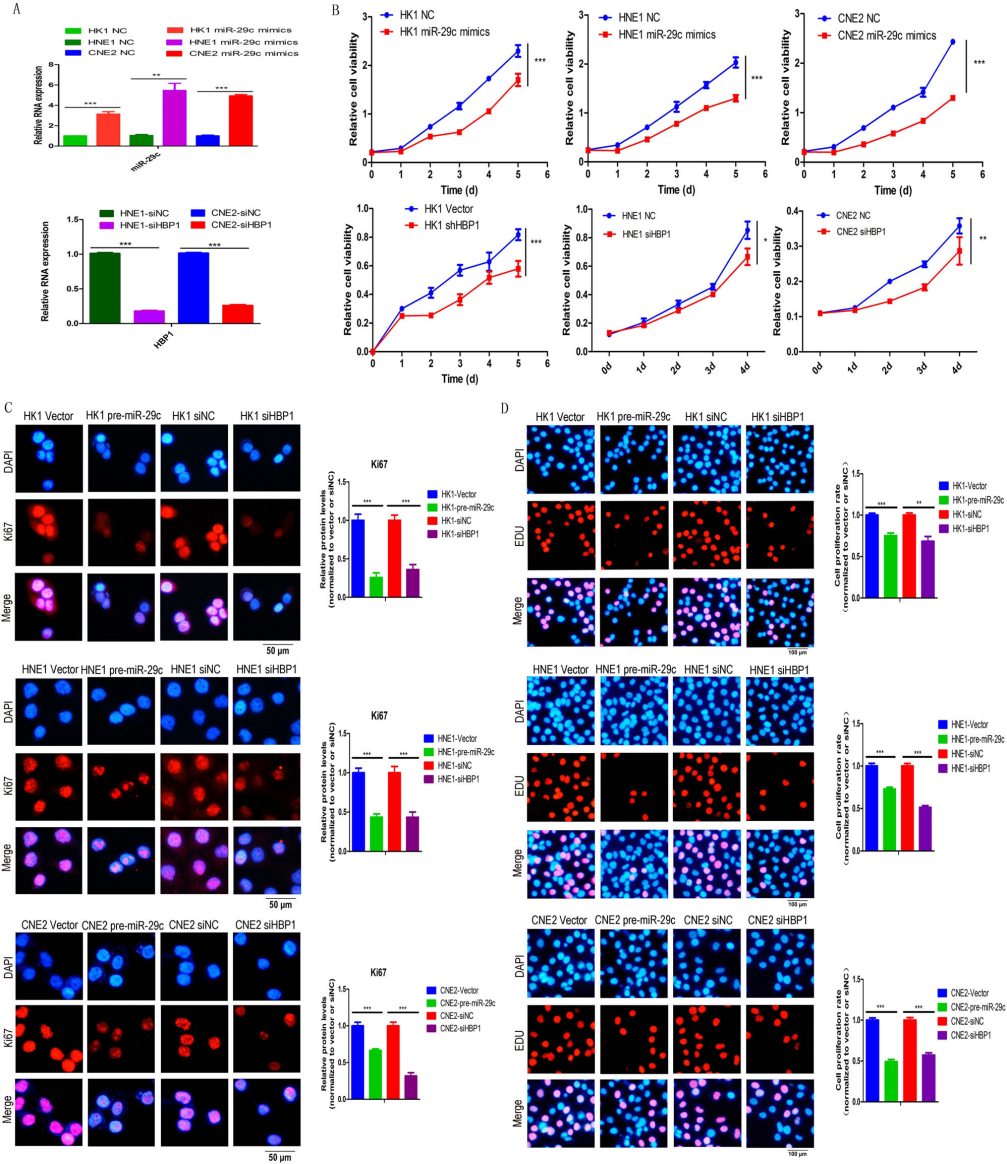
HBP1 facilitates NPC cell proliferation in vitro **a** Upper: RT-qPCR analysis for miR-29c miRNA levels in HK1, HNE1, and CNE2 cell lines after transfection with miR-29c mimics or NC mimics. Lower: RT-qPCR analysis for HBP1 mRNA levels in HNE1 and CNE2 cell lines after transfection with siHBP1 and siNC. **b** Upper: CCK-8 assays analysis for HK1, HNE1, and CNE2 cells transfected with miR-29c mimics or NC mimics. Lower: CCK-8 assays analysis for HK1, HNE1, and CNE2 cells transfected with siHBP1 or siNC. **c** Ki67 cell proliferation assay for HK1, HNE1 and CNE2 cell lines after transfection with pre-miR-29c or vector, siHBP1 or siNC. The grouped graphs are the semiquantitation for Ki67 protein. *t* test analyses. ***p < 0.001. Scale bars, 50 μm. **d** EDU assay for HK1, HNE1, and CNE2 cell lines after transfection with pre-miR-29c or vector, siHBP1 or siNC. The grouped graphs are analyzed for the cell proliferation rate. *t* test analyses. ***p < 0.001; **p < 0.01. The cell proliferation rate was normalized with the ratio for the EDU-stained cells to the nucleus-stained cells. Scale bars, 100 μm

To illustrate whether HBP1 exerts biological functions on NPC cells, we designed specific siRNAs against HBP1 and transfected into three NPC cell lines (HK1, HNE1, and CNE2). MiR-29c miRNA levels increased after transfection with mimics and HBP1 mRNA levels decreased after transfection with siRNA (Fig. 4a). Note that ectopic expression of miR-29c and suppression of HBP1 both resulted in significant inhibition in HK1, HNE1, and CNE2 cell proliferation, as determined by CCK-8 assay (Fig. 4b). To further demonstrate whether HBP1 plays a role in promoting rather than inhibiting NPC cell growth, we performed Ki67 and EDU assays to detect cell proliferation when HBP1 knockdown. Ki67, a nuclear cellular proliferation-associated marker expressed in all active stages of the cell cycle, which can act as a cell proliferation marker during G1, S, G2, and mitosis phases^24^. EDU (5-ethynyl-2′-deoxyuridine) is a thymidine analog which can replace thymine (T) through inserting into replicating double-strand DNA in proliferating cells^25^. Ectopic expression of miR-29c in HK1, HNE1 and CNE2 cell line decreased Ki67 protein expression and EDU-staining cells, which suggested that the cellular proliferation rate was reduced. HBP1 knockdown also decreased Ki67 protein expression and EDU-staining cells in NPC cells (Fig. 4c, d).

**Fig. 5:**
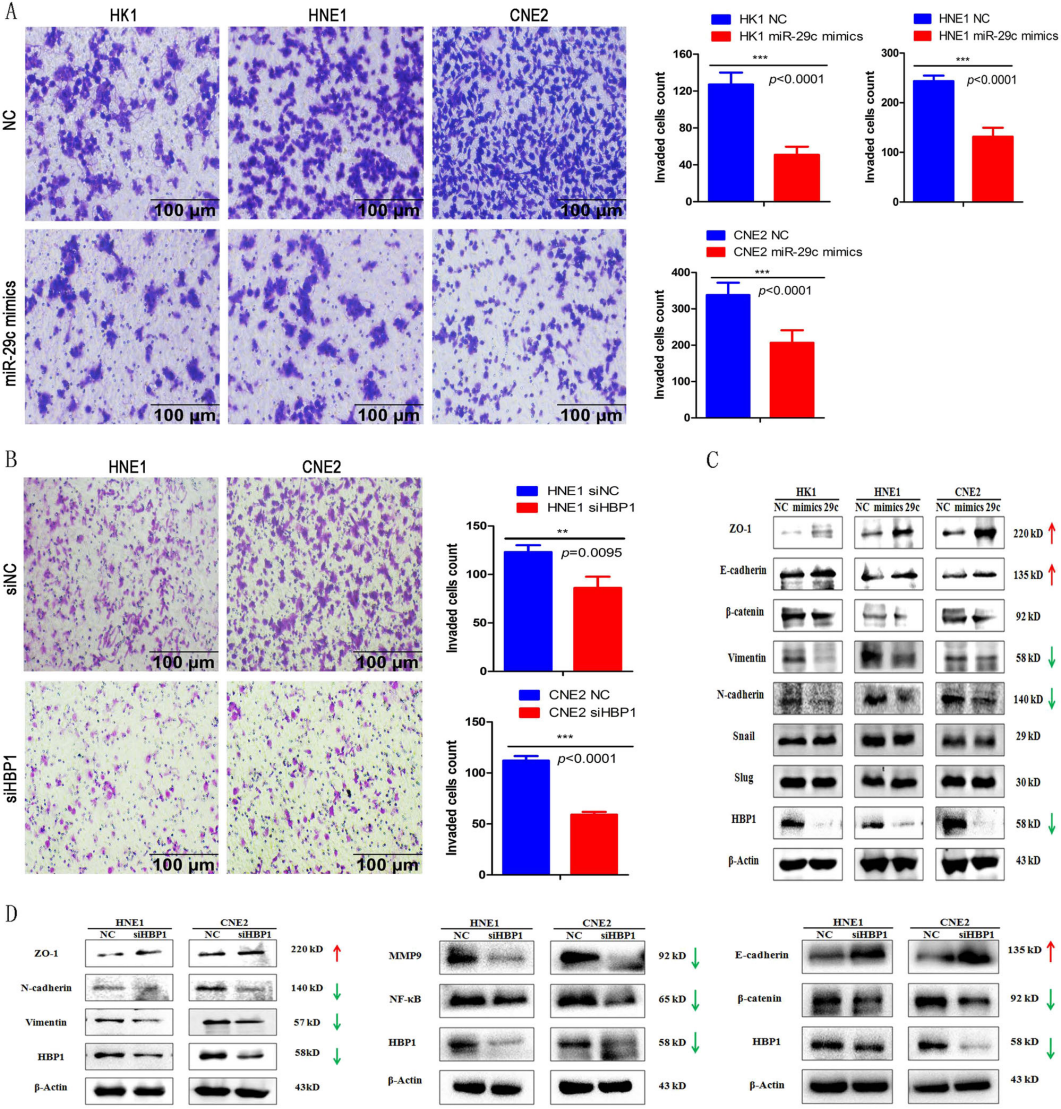
HBP1 promotes NPC cell invasion in vitro **a** Re-expression of miR-29c inhibited HK1, HNE1, and CNE2 cells invasion in vitro as measured by trans-well assay. Right panel: Statistical analysis for the invaded cells, ***p < 0.001. Scale bars, 100 μm. **b** HBP1 knockdown inhibited HNE1 and CNE2 cells invasion in vitro as measured by a trans-well assay. Right panel: Statistical analysis for the invaded cells. *p* = 0.0095 and *p* < 0.0001. Scale bars, 100 μm. **c** Western blotting for invasion-associated molecules (N-cadherin, Vimentin, and β-catenin) and inhibitory molecules (ZO-1 and E-cadherin) in HK1, HNE1, and CNE2 cells after transfection with miR-29c mimics or NC. **d** Western blotting for invasion-associated molecules (N-cadherin, Vimentin, β-catenin, MMP9, NF-κB) and inhibitory molecules (ZO-1 and E-cadherin) in HNE1 and CNE2 cells after transfection with siHBP1 or siNC

To detect roles of HBP1 in cell invasion, we conducted transwell assays in NPC cells. As expected, miR-29c ectopic expression significantly reduced invasion in the HK1, HNE1, and CNE2 cell lines (Fig. 5a). It is intriguing that HBP1 knockdown also reduced the number of invading cells among HNE1 and CNE2 cells (Fig. 5b). The western blotting results showed that ectopic expression of miR-29c suppressed cellular invasion-promoted proteins expression, Vimentin and N-cadherin, while increased invasion-suppressed proteins expression, ZO-1 and E-cadherin (Fig. 5c). Surprisingly, HBP1 knockdown up-regulated ZO-1 and E-cadherin protein expression and down-regulated invasive proteins, including N-cadherin, Vimentin, β-catenin, MMP9 and NF-κB (Fig. 5d).

Above results confirm that HBP1 was highly expressed and played a role in promoting cellular proliferation in NPC cells, then we explored whether HBP1 mutations was present in NPC cells. The blast results of HBP1 cDNA cloned from NP69, HNE1, HK1, and CNE2 cell lines (Fig. S1e) further confirmed that there is no mutation for HBP1 in NPC cells via the NCBI Web Blast Tool (https://blast.ncbi.nlm.nih.gov/Blast.cgi) (data not shown).

### HBP1 knockdown suppresses NPC cell proliferation through G1-S phase arrest rather than apoptosis

**Fig. 6:**
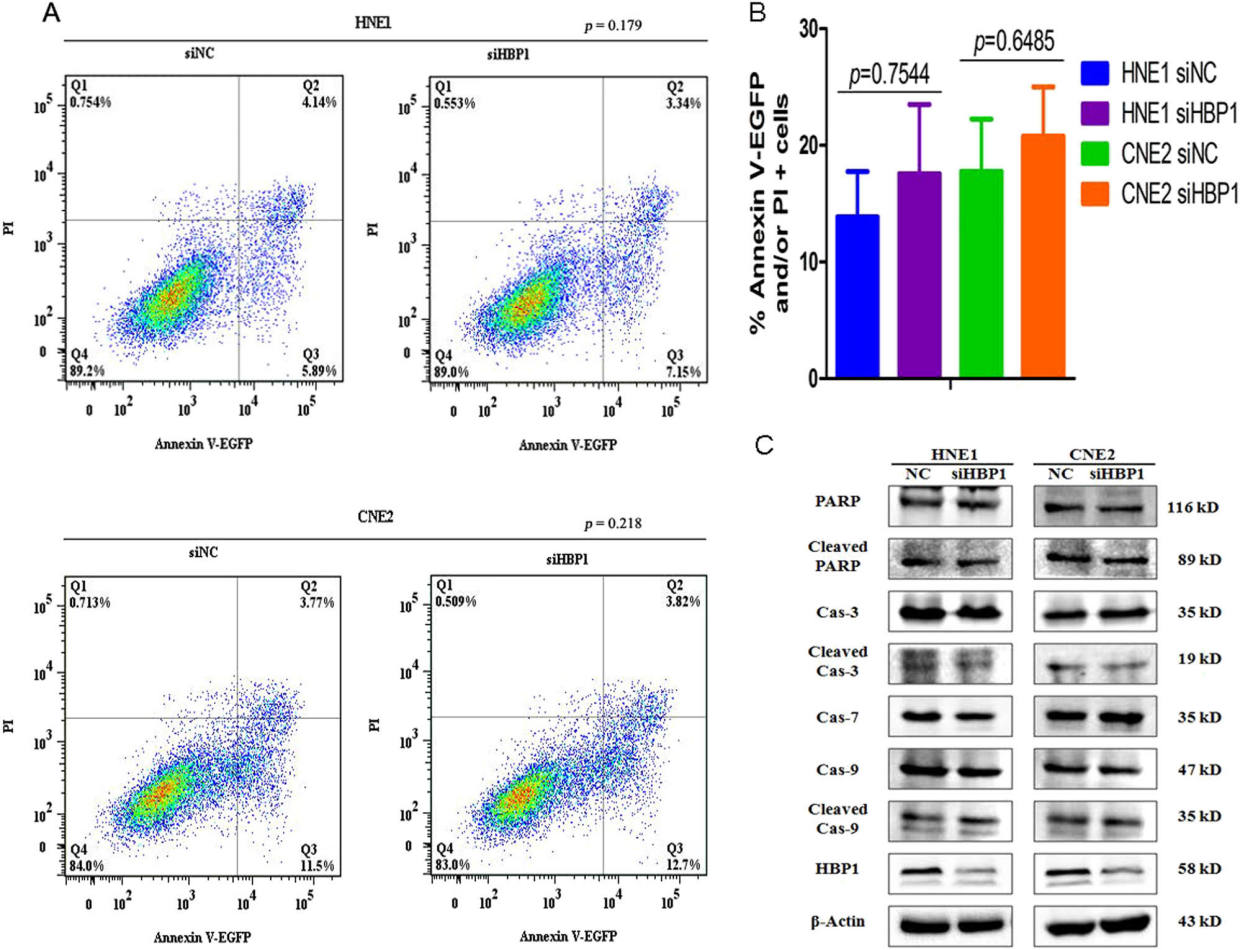
HBP1 does not affect NPC cells apoptosis **a** Cell apoptosis analysis for HNE1 and CNE2 cells after transfection with siHBP1 or siNC for 48 h by Annexin V-EGFP and PI (propidium iodide) co-staining assays, *p* = 0.179 and *p* = 0.218. **b** Percentage of Annexin V-EGFP and/or PI staining-cells as above (**a**). **c** Western blotting analysis for total and cleaved PARP, caspase 3 (Cas-3), and caspase 9 (Cas-9). β-actin was used as an internal reference

The promoting effects of HBP1 on NPC proliferation cells might be partly attributed to changes in cell growth regulation, such as cellular senescence, apoptosis or cell cycle arrest. Thus, we performed cellular apoptosis and cell cycle assays in the HNE1 and CNE2 cell lines by flow cytometry (FCM) analysis. Apoptosis assays data indicated that the proportion of Annexin V-EGFP-(stains apoptotic cells) and propidium iodide-(PI, stains necrotic and late apoptotic cells) positive cells did not change significantly after HBP1 knockdown in the HNE1 and CNE2 cells (Fig. 6a, b). The western blotting results suggested that the total and cleaved caspase 3 (Cas-3), caspase 7 (Cas-7), caspase 9 (Cas-9), and PARP proteins levels were not significantly altered (Fig. 6c).

**Fig. 7:**
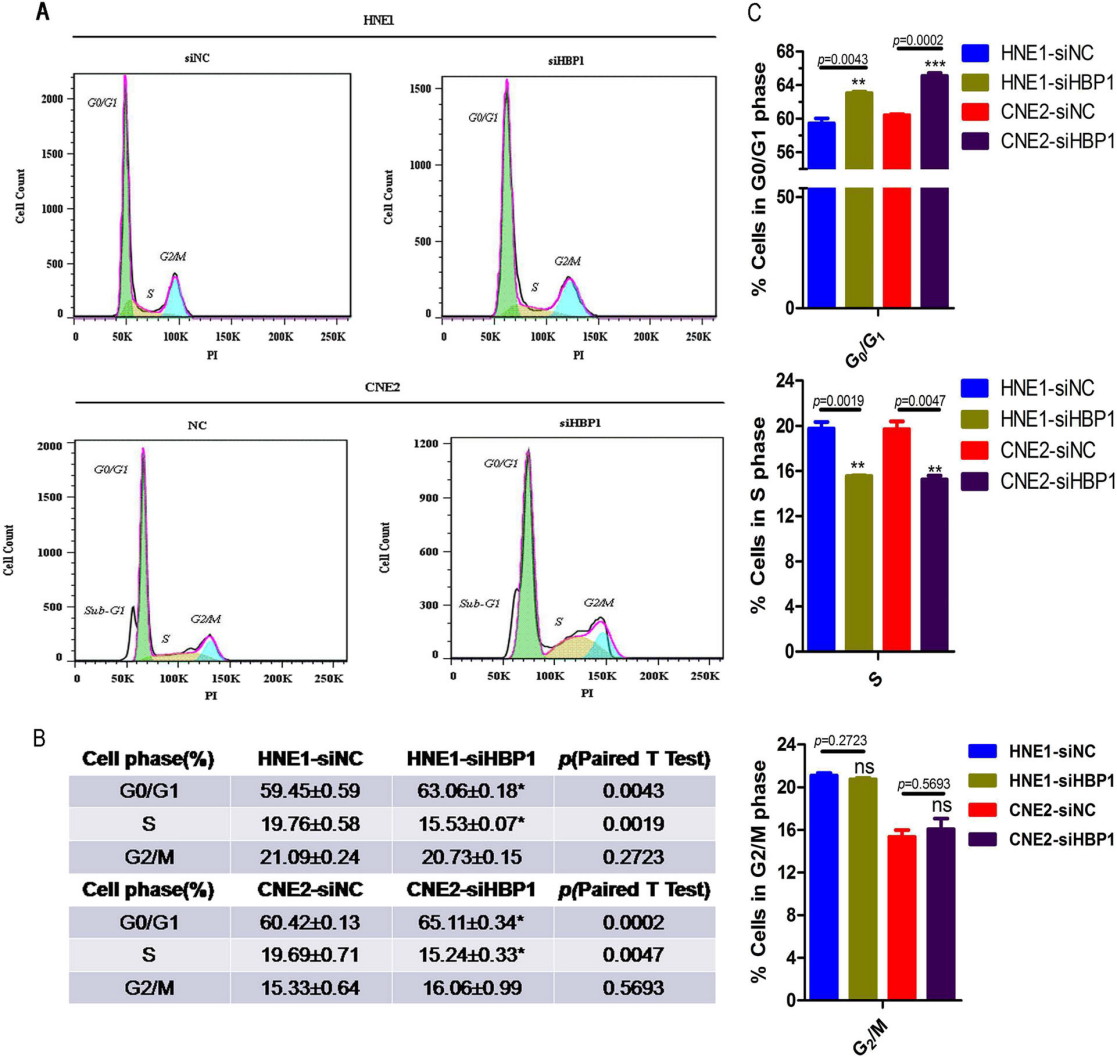

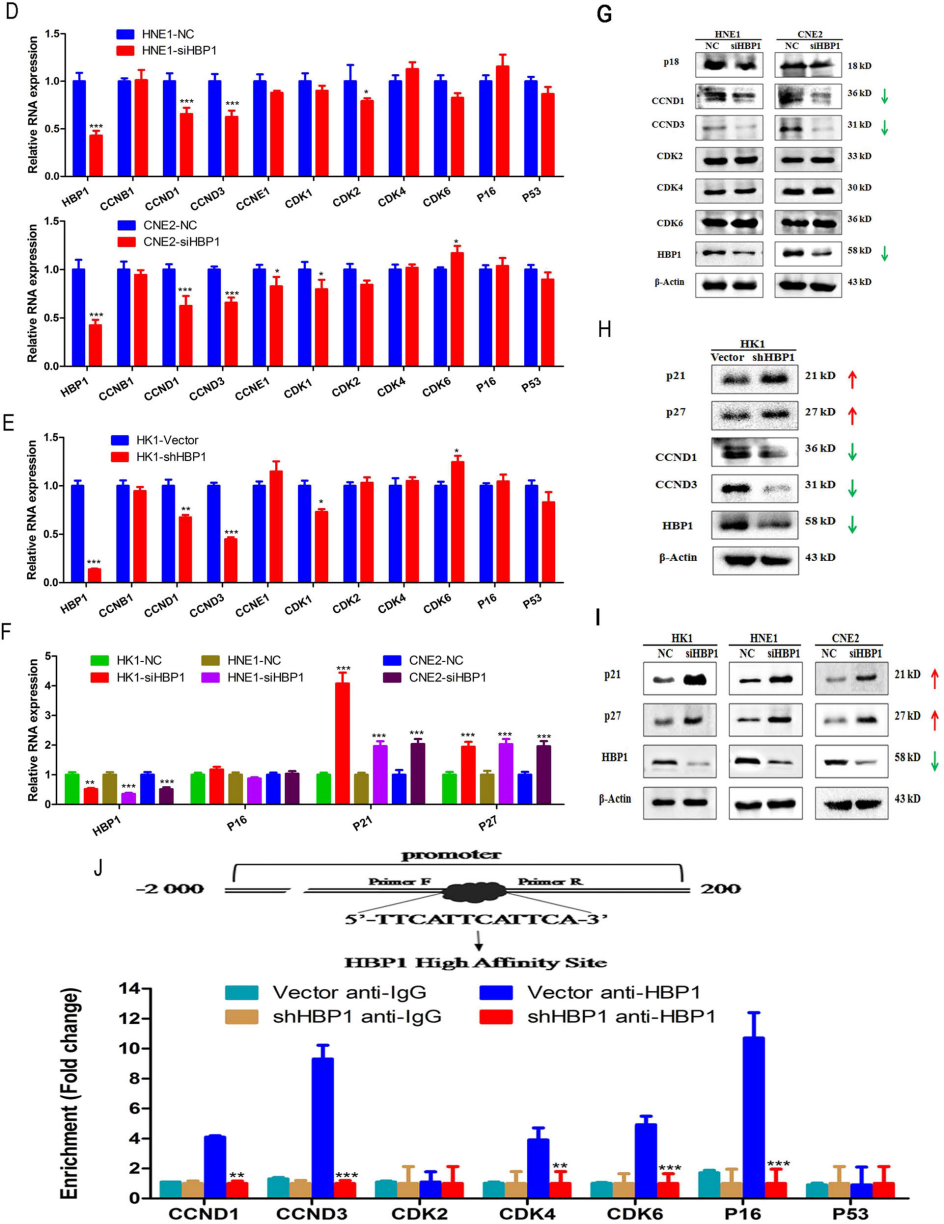
HBP1 expedites the progression from G1 to S phase in NPC cell lines **a** Cell cycle analysis for the HNE1 and CNE2 cell lines transfected with siHBP1 or siNC using PI staining. **b** Cell cycle distribution in the G0/G1, S and G2/M phases as above **a**. **c** Statistical analysis for the G0/G1, S and G2/M phases as above (**b**). **d**–**f** RT-qPCR assays for the cell cycle-related molecules (CCNB1, CCND1, CCND3, CCNE1, CDK1, CDK2, CDK4, CDK6, p16, p21, p27, and p53) after HBP1 knockdown in HEN1, CNE2, and HK1 cells lines. **g**–**i** Western blotting for the cell cycle-related molecules (p18^INK4C^ (p18), p21^Waf1/Cip1^ (p21), p27^Kip1^ (p27), cyclin D1 (CCND1), cyclin D3 (CCND3), CDK2, CDK4, and CDK6) after HBP1 knockdown in HNE1, CNE2, and HK1 cells lines. β-Actin was used as an internal reference. **j** Upper: Diagram of the HBP1 high affinity site to promoters and location of the primers. Bottom: ChIP-qPCR of HBP1 association in HK1 cells. ChIP was carried out with HBP1 antibody, with mouse monoclonal antibody IgG as control. IgG was used for normalization

While cell cycle analysis revealed that HBP1 knockdown increased the number of cells in G1 phase accompanied with decreased cells in S phase (Fig. 7a–c). The RT-qPCR results showed that HBP1 knockdown induced p21 and p27 expression, and simultaneously inhibited cyclin D1 (CCND1) and D3 (CCND3) mRNA expression in the HNE1, CNE2, and HK1 cell lines (Fig. 7d–f), which was consistent with the protein expression variations (Fig. 7g–i). These data demonstrate that HBP1 promotes cell proliferation may through, in part, elevating CCND1 and CCND3 proteins levels and inhibiting CDKI activation, p21 and p27.

HBP1 is located on Chromosome 7 at 107,168,290–107, 203,200. Its full-length mRNA is 2749 bp, and its CDS sequences are 1575 bp, which encodes a 514 amino acid protein (Figs. S3a–b)^26^. Gene Ontology (GO) annotations^27^ show that the processes, functions and components of HBP1. HBP1 was involved in transcription regulation, cell cycle arrest and the Wnt signaling pathway, and functions at multiple levels, including by binding DNA, RNA or proteins (Figs. S3c). HBP1 is mainly located in the cell nucleus and might exert biological functions in nucleus, thus, we determined whether it could bind to cell cycle molecules promoters via ChIP. HBP1 binds to the p16^INK4A^ promoter has demonstrated by previous studies; thus, the p16^INK4A^ promoter levels were regarded as a positive molecular contrast^15,28^. In ChIP assays, the bound promoter DNA was magnified and the levels are normalized to the percentage of the input DNA by q-PCR. Indeed, when HBP1 was knocked down, we detected at least 4-fold decrease in HBP1 enrichment to the CCND1, CCND3, CDK4, CDK6, and p16^INK4A^ promoters (Fig. 7j). These data show that HBP1 may bind to the CCND1 and CCND3 promoters and induce its expression in cell cycle progress, HBP1 also bound to the p16^INK4A^, CDK4 and CDK6 promoters, while did not affect their RNA and protein levels, it may explain, in part, HBP1 bounds to p16^INK4A^ and CDK4 promoters that competitively inhibits the effects of p16 inhibiting CDK4, thus, the effects of CCND1-CDK4 and CCND3-CDK6 complexes may be promoted and downstream cell cycle effectors may be activated.

### HBP1 knockdown restrains NPC xenograft tumor growth and metastasis in vivo

**Fig. 8:**
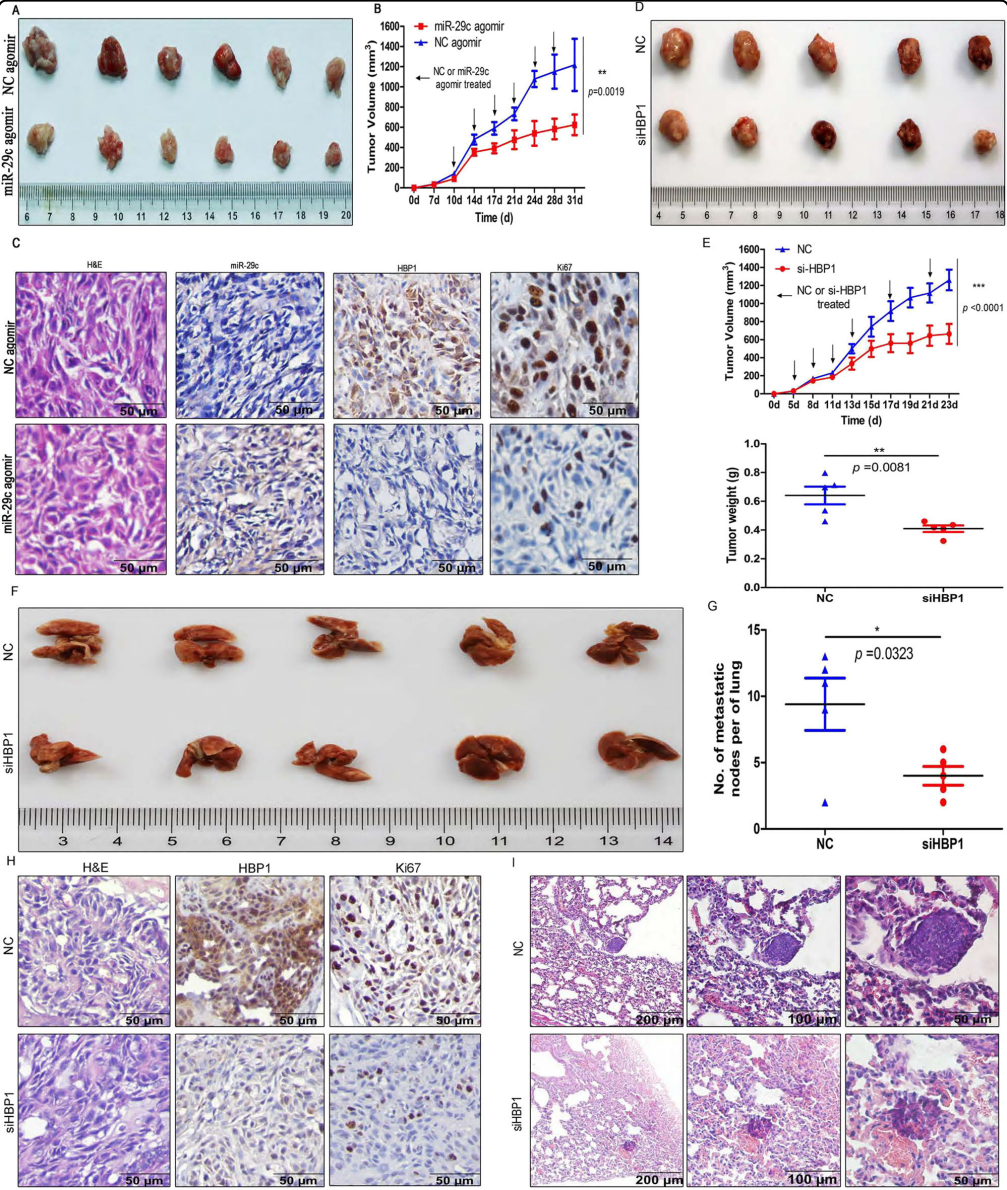
HBP1 knockdown blocks NPC xenograft growth and metastasis in vivo **a** Xenograft tumors in nude mice injected with miR-29c agomir or NC agomir were removed (31 days) after their initial transplantation in HK1 cells. Scale bars, 0031 cm. **b** Tumor volume curve for HK1 cells transplanted into nude mice with miR-29c or NC agomir therapy. (*p* = 0.0019). **c** Paraffin sections were stained with H & E and in situ hybridization for detecting miR-29c expression levels. Immunohistochemistry for HBP1 and Ki67 protein levels in miR-29c agomir or NC agomir-treated group tumor tissues. Scale bars, 100 μm. **d** Tumor growth observed over time showed a reduction in the group with siHBP1 treatment compared with NC treatment. Scale bars, 1 cm. **e** Top panel: Tumor growth curve of xenografts in nude mice as above (**d**), *p* < 0.001. Bottom panel: Tumor weight of xenografts in nude mice as above **d**, *p* = 0.0081. **f** Metastasis in the lungs from tumor-bearing nude mice treated with siHBP1 or NC. Scale bar, 1 cm. **g** Statistical analysis for the metastatic nodes in nude mice as above (**f**). **h** H & E and immunohistochemistry analysis for paraffin sections of nude mice from tumor xenografts. **i** H & E staining for paraffin sections from nude mice lungs tissues. Scale bars, 200 μm, 100 μm and 50 μm, respectively

To verify our results in vivo, 2′-O-methyl-conjugated 5′-cholesterol (2′Ome-5′Chol) modified miR-29c agomir was used as a simulative miRNA to increase miR-29c levels in nude mice (Figs. S4a–c). The tumors grew in the miR-29c agomir group were smaller than those grew in the NC agomir group, which indicated that miR-29c ectopic expression exerted an anti-tumor effect on NPC cells in vivo (Fig. 8a). Tumor growth was remarkably inhibited by miR-29c agomir, and the final volume of the tumors in the NC agomir group was ~2 times larger than that in miR-29c agomir group (Fig. 8b). In situ hybridization data showed that miR-29c was more highly expressed in tumor tissues from miR-29c agomir-injected mice than NC agomir-injected mice (Fig. 8c). IHC staining indicated that HBP1 expressed lower levels in the tumor tissues from the miR-29c agomir-injected mice and higher HBP1 expression levels than in the NC agomir group; additionally, the cells of cellular proliferative nuclear antigen Ki67-stained were decreased (Fig. 8c). These data demonstrate that HBP1 knockdown via miR-29c agomir intratumoral injections inhibited xenograft growth in vivo, which was in accordance with the results that HBP1 expression inhibition decreased NPC cells growth and proliferation in vitro.

Furthermore, 2’Ome-5’Chol-modified si-HBP1 or a negative control (siNC) was prepared for rescue experiments in vivo (Figs. S4b, c). The data indicated that the size of siNC-treated mice doubles than siHBP1-treated mice, and the tumor weight was 1.5-fold increased (Fig. 8d, e). Regarding HK1 cells invasiveness and metastasis, white nodules were observed on the lung surface in both siNC-traeted and siHBP1-treated nude mice (Fig. 8f). Similarly, the number of lung metastatic nodes of siHBP1-treated mice (9.4 ± 2.0 nodules) significantly reduced compared to the siNC-treated mice (4.0 ± 0.7 nodules) (Fig. 8g). IHC indicated that both HBP1 and Ki67 stained cells were reduced in the siHBP1-treated nude mice (Fig. 8h), which suggested that HBP1 knockdown decreased NPC tumor growth. H & E staining of the lung paraffin sections showed that HBP1 promoted HK1 cells transferring to the lungs in nude mice (Fig. 8i). These results further confirmed that HBP1 not only increased proliferation but also cell migration in xenograft-bearing mice in vivo.

**Fig. 9:**
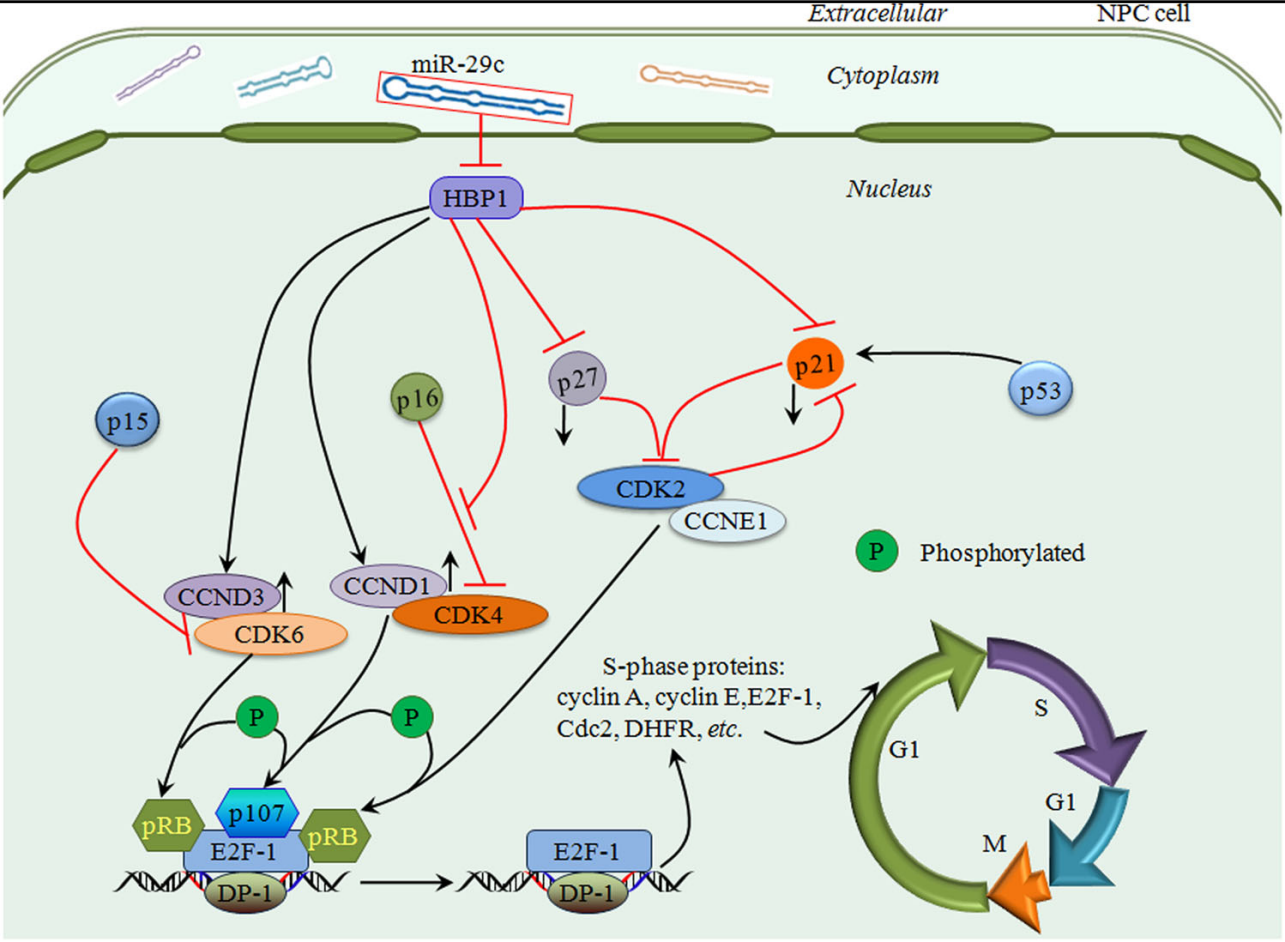
A model for the HBP1-dependent regulation of cell cycle in NPC cells MiR-29c inhibited HBP1 expression by targeting the HBP1 3’UTR. However, HBP1 expedited the G1 to S phase progression for promoting cell growth and proliferation by inhibiting p21 and p27 and simultaneously inducing CCND1 and CCND3 expression. On one hand, HBP1 binds to the CCND1 and CCND3 promoters and activates their transcription. HBP1 also bind to the p16^INK4A^, CDK4, and CDK6 promoters but does not affect their expression, it may explain, in part, HBP1 bounds to p16^INK4A^ and CDK4 promoters that competitively inhibits the effects of p16 inhibiting CDK4, thus, the effects of CCND1-CDK4 and CCND3-CDK6 complexes may be promoted and downstream cell cycle effectors may be activated. Subsequently, it facilitates CCND1-CDK4, CCND3-CDK6 and promotes the phosphorylation of p107 and pRB, respectively. On the other hand, HBP1 binds to the p21^Waft/Cip1^ promoter and inhibits its transcription, thus inhibiting the effect of CDK2 due to p21^Waft/Cip1^ was removal. HBP1 also inhibits p27^Kip1^ expression and in turn the CDK2-CCNE1 complex activates the phosphorylation of pRB. Hereon, the transcription of S-phase related genes were activated including cyclin A, cyclin E, E2F-1, DP-1, etc., which accelerates the G1-to-S phase transition

Based on our findings, we have built a model regulatory network for HBP1 and miR-29c. In this model, HBP1 binds to the CCND1 and CCND3 promoters and activates their transcription, HBP1 also binds to p16^INK4A^ promoter, but does not change its expression, though it does remove the inhibitory effect of p16 on CDK4, thus, CCND1-CDK4 and CCND3-CDK6 complexes facilitate the phosphorylation of p107 and pRB, respectively. Moreover, HBP1 binds to the p21Waft/Cip1 promoter and inhibits its transcription, which inhibits the effect of CDK2 due to the removal of p21Waft/Cip1. HBP1 also inhibits p27^Kip1^ expression; in turn, the CDK2-CCNE1 complex activates the phosphorylation of pRB. Hereon, S-phase genes transcriptions are activated, including that of cyclin A, cyclin E, E2F-1, DP-1, etc, which expedites the G1/S transition (Fig. 9).

## Discussion

As a transcription factor, HBP1 has diverse functions in various biological processes. HBP1 is widely expressed in multiple tissues and myogenic and adipocyte cell lines and is directly correlated with cell differentiation^11–13,29^. HBP1 also participates in promoting premature senescence^15,30,31^. HBP1 was reported to be a tumor repressor in several cancers including prostate cancer, non-small cell lung carcinoma (NSCLC) and breast cancer^17,32–34^, in which it also induced cell cycle arrest and promoted cell apoptosis by inhibiting Wnt/β-catenin pathway signal transduction^18,20,21^. In human non-small cell lung cancer (NSCLC), low HBP1 protein expression is correlated with a poor prognosis in 82 NSCLC patients by immunohistochemical analysis; it was also associated with lymph node metastasis and early TMN stages^32^. HBP1 had low expression in leukemic myeloid cells^29^, invasive breast cancer^33^ and prostate cancer^17^. We also have tested HBP1 protein in non-NPC cells by western blotting, which is lowly expressed in liver cancer, breast cancer and ovarian cancer cells (Fig. S1f). However, in our study, HBP1 mRNA and protein levels was highly expressed in NPC tissues and cell lines (Fig. 1 and Fig. S1f). All of the experimental results show that HBP1 positively promoted NPC cells proliferation and invasion and was negatively correlated with NPC patient’s prognosis.

According to previous studies, there were three HBP1 regulatory mechanisms in the cell cycle^35^. In differentiated hepatocytes from HBP1 transgenic mice, cyclin E and kinase activity and immediate-early gene expression were significantly decreased, leading to a lengthened G1 phase and delayed S phase^36^. P38 kinase activation promotes its downstream molecules p21^Cip^ and HBP1, which suppressed the cell cycle, and cyclin D1, thus facilitating the cell cycle and promoting G1 progression^37^. In neural stem/progenitor cells, HBP1 inhibits cyclin D1 and limits the G1/S transition; this finally inhibits cell cycle progression, which is a key mechanism of neuronal differentiation concerning the proper timing of neural stem cells (NSCs). However, we detected that HBP1 knockdown arrested cells in the G1 phase and simultaneously inhibited the transition from G1-phase to S-phase in NPC cells. We also found that HBP1 knockdown contributed to increasing p21^Waf1/Cip1^ and p27^Kip1^ proteins expression, but decreased cyclin D1 and D3 expression in HNE1, HK1 and CNE2 cells (Fig. 7g–i). Nevertheless, we found that the expression was unchanged in cyclin-dependent kinase, CDK2, CDK4 and CDK6 (Fig. 7g). This completely different role for HBP1 in the development of malignant tumors demonstrated that the context of up-stream molecules of HBP1 in different cancers was not different, though this did lead to differences in the roles of HBP1 and the molecular mechanism.

As reported previously, HBP1 bound to specific sequences in endogenous p47phox and inhibited its expression, which lead to decreased superoxide levels and proliferation^14^. In prostate cancer, HBP1 directly targeted the MIF promoter and inhibited its transcription, which remarkably blocked cell growth and invasion^38^. In Ras-mediated cell environments, HBP1 directly bound to p16^INK4A^ and elevated its expression, which subsequently caused premature senescence. This also indicated that HBP1-induced transcriptional regulation was important for both premature senescence and tumorigenesis^15^. It was confirmed that histone acetyltransferase p300 and CREB-binding protein (CBP) could acetylate HBP1 protein in human lung fibroblasts cells, and that p300/CBP was recruited to the p16^INK4A^ promoter by HBP1 to increase p16 transcription^28^. HBP1 bound to TCF4 and inhibited the transcription of EZH2, which led to a decrease H3K27me3 of p21^Waft/Cip1^ promoter, thus leading to premature senescence and the inhibition of tumorigenesis^31^. The ChIP assay showed that HBP1 bound to the CCND1, CCND3, and p21 promoters. HBP1 also binds to the p16^INK4A^ promoter and may prevent its suppressive role on CDK4; this allows CDK2-CCNE1, CDK4-CCND1, CDK6-CCND3 complexes formation and then participates in the G1 to S phase process (Fig. 7j). On one hand, the mechanism of HBP1 inhibited p21 and p27 expression might through bound their silencers, and the activation of CCNE1-CDK2 complex might be regulated by epigenetic modification, both of which will be further explored in our next study. HBP1 knockdown not affects CDK2, CDK4 and CDK6 expression; therefore, we next will detect the phosphorylation status of those CDKs. On the other hand, p21 could inhibit the kinase activity of complexes contain CDK2, CDK4, and CDK6, p27 could inhibit the activity of CCNE1-CDK2 and cyclin D-CDK4, which may explain, in part, why the process of G1 phase to S phase accelerated by degradation of p21 and p27 induced by HBP1.

Although we are currently unable to thoroughly identify the HBP1 mechanisms regulating G1-S processes and cell invasion, our findings have verified the theory that identical gene may show different and even reverse functions in different contexts and microenvironments through different regulatory networks. In summary, our study demonstrates that miR-29c directly targeted HBP1 and down-regulated its expression. HBP1 promoted NPC cell proliferation and invasion, while miR-29c expression inhibited cell proliferation in vitro and in vivo. Additionally, HBP1 knockdown suppressed NPC cell proliferation by arresting cells in G1-phase rather than by affecting cell apoptosis. HBP1 promoted the proliferation in NPC cells by increasing CCND1 and CCND3 expression via directly interacting with their promoters, and decreasing p21^Waf1/Cip1^ and p27^Kip1^, that the CDK2-CCNE1, CDK4-CCND1, CDK6-CCND3 complexes would be activated to promote G1 to S phase process. HBP1 may also promote invasion by down-regulating E-cadherin and ZO-1 expression and up-regulating β-catenin, Vimentin, MMP-9, N-cadherin, and NF-κB expression. Furthermore, miR-29c inhibited NPC tumor growth while HBP1 expedited tumorigenesis in vivo.

## Materials and methods

### Tissue sample

Sixty one clinical specimens (31 NPC tissues and 30 para-tumor tissues) were collected from the Pathology Department of the Second Xiangya Hospital of Central South University (Changsha, China). The NPC tissues were paraffin-embedded with EG1160 Paraffin Embeddly Center (Leica, Germany), and then sliced using RM2245 Semi-Automated Rotary Microtome (Leica, Germany). Paraffin sections conducted to immunohistochemistry and in situ hybridization.

### Cell culture

HEK293 cells were cultured in DMEM medium (Gibco, Grand Island, NY, USA) with 10% fetal bovine serum (FBS) (Gibco, Grand Island, NY, USA). Normal nasopharyngeal epithelial cell line, NP69, was a gift obtained from Professor Sai Wah Tsao of the Department of Anatomy of University of Hong Kong, maintained in keratinocyte/serum-free medium (Invitrogen, Carlsbad, USA) with the growth factor supplements (Life Technologies, Gaithersburg, MD, USA). HNE1, HK1, CNE2, C666–1 cell lines were cultured in RPMI 1640 medium (Gibco, Grand Island, NY, USA) with 1% Penicillin-Streptomycin Solution (BI, Kibbutz Beit-Haemek, Israel) and 10% FBS at 37 °C in 5% CO_2_ incubator. Cells were dissociated with trypsin EDTA solution A (BI, Kibbutz Beit-Haemek, Israel) and frozen in 90% FBS with 10% DMSO (Sigma-Aldrich, St. Louis, MO, USA).

### Bioinformatics analysis

A NPC gene microarray data from the platform of Affymetrix Human Genome U133 Plus 2.0 Array (HG-U133_Plus_2) in GEO Datasets (GEO accession: GSE12452). We used Multiple Array Viewer to conduct a significant analysis by microarray software that analyzes the microRNAs differently expressed between normal nasopharyngeal epithelium and NPC tissue from GSE12452 data (see Table S1 and S2)^39^. The miRNAs with significant different delta value of 1.5–5 fold difference were selected out for further analysis. Targeted genes of miR-29c were predicted by miRanda^40^ and TargetScan^23^ programs (see Table S3, the Total Context + + score was set at <−0.7).

### Immunohistochemistry

The paraffin sections staining and evaluation were performed as previously described^10^. The dilution ratio of anti-HBP1 (Millipore, Billerica, MA, USA) and Ki67 (BBI Life Sciences, Shanghai, China) was 1: 200 and 1: 300, respectively. All sections were scored independently by three researchers according to the clinical data and clinicopathological features. On the high magnification, the comprehensive staining intensity and the proportion of positive cells was used for semi-quantitative determination. Dying color was scored as 0 for no staining, 1 for light yellow, 2 for light brown and 3 for brown. Proportion of positive cells was graded as score 0, <5%; score 1, 5–25%; score 2 points, 26–50%; score 3, 51–75%; and score 4, >75%. Two scores multiplied together and the total score divided into four ranks: 0 for negative expression (−), 1 to 4 for weak expression (+), 5 to 8 for middle positive expression (+ +), and 9 to 12 for strong positive expression (+ + +).

### HBP1 3′UTR (untranslated region) constructs

The consensus sequence of miR-29c targets for HBP1 mRNA was predicted by the TargetScan and miRanda (see Table S2 and Fig. S1) programs. The wild type and mutant seed sequences of HBP1 3′UTR miR-29c targeting were synthesized by Invitrogen (detailed sequence see Table S4), which was cloned into the *Hind* III/*Mlu I* site of pMIR-Report (TAKARA, Japan) and transformed into *Ecoli* JM109. HBP1 3′UTR wild-type or mutant constructs vector were extracted by E.Z.N.A.^®^ Endo Free Plasmid Mini Kit II (OMEGA, Norcross, GA, USA) followed by the manufactures’ instructions. The inserts were sequenced from Biosune (Shanghai, China).

### Pre-miR-29c and shHBP1 constructs

Full length of pre-miR-29c sequences was obtained from NCBI, shHBP1 sequences was designed by Thermo Fisher Scientific BLOCK-iT™ RNAi Designer, and both sequences were synthetized by Invitrogen (primer sequence was listed in Table S4). The forward and reverse primers of pre-miR-29c and shHBP1 were denatured in 95 °C for 10 min and annealed by cooling in room temperature. Pre-miR-29c and shHBP1 sequences were cloned into the *BglII*/*Hind III* site of pSUPER vector (OligoEngine, WA, USA) and transformed into *Ecoli* JM109. We then digested the pSUPER-shHBP1 vector with *BamHI* and *ClaI*, and the linear fragment which contained shHBP1 sequences were inserted into the pLVTH (OligoEngine, WA, USA) lentivirus vector which was digested with the same restriction enzymes (*BamHI* and *ClaI*) and then transformed into *Ecoli* JM109 for conservation and following experiments. Whole HBP1 cDNA primers (Table S4) was synthesized by Invitrogen Company, and cDNA was amplified by RevertAid Reverse Transcriptase (Thermo Scientific, MA, USA). All constructs region were sequenced using consensus primers according to vector’s instruction in BioSune (Co., Ltd, Shanghai, China).

### Lentivirus production and infection

Vector and shHBP1 constructs were packed into HEK 293 FT cells co-transfected with virus packaging plasmids including DR 8.47, PMD2G and REV (Addgene, MA, USA). Virus supernatant was collected after 48 h transfection, centrifuged, filtered and packed in −80 °C cryopreservation. 2.0 × 10^4^ HK1 and CNE2 cells were seeded in 6-well dish, and then infected with 2 ml virus supernatant and 2 μl infection promoting reagents Polybrene (Santa Cruz Biotechnology, CA, USA). Cells were observed under fluorescence microscope after 48 h infection. Infected cells were cultured until the cell density reaches to 90%, and the positive cells were sorted by Flow cytometry and cultured for further experiments.

### Luciferase activity assay

HEK-293T (1 × 10^5^) cells were seeded into 24-well plates one day before transfection. When the cells grew to about 70% density, both pSUPER-Con and pSUPER-pre-miR-29c were co-transfected with pMIR-Report-HBP1 3′UTR wide-type or mutant constructs on the condition of transfection with 100 ng Renilla vector. Luciferase activity was measured with dual luciferase assay (Promega, WI, USA) 48 h after transfection. The luciferase activity was normalized to the Renilla luciferase activity.

### SiRNA transfection for HBP1

SiHBP1 (5′-UAC CUC AGA CAU ACC AGA ATT −3′) and negative control (NC) siRNA duplexes were synthesized by GenePharma (Shanghai, China). Transfection was performed with Lipofectamine 2000 Reagent (Invitrogen) according to the manufacturer’s protocol. The final concentration of siRNA and NC was 40 nM; pre-miR-29c and vector were 3 μg in 2 ml medium. Total RNA was extracted and the expression of miR-29c or HBP1 was tested by RT-qPCR. Protein extraction and western blotting assay were performed 48 h after transfection.

### In situ hybridization

In-situ hybridization was performed to detect miR-29c expression in NPC tissues specimens and NPC cell line (HK1, HNE1 and CNE2). The synthetic miR-29c Digoxin tag probe sequences is 22 oligonucleotides, 5′-TAG CAC CAT TTG AAA TCG GTT-3′, and synthesized by Sangon Biotech (Shanghai, China). Before experiment, cells were transfected with siNC and siHBP1, or vector and pre-miR-29c, respectively, cells were collected by trypsinization and seeded upon glass slide in 6-well plates for 20 h. In situ hybridization was performed according to the manufacture’s protocol. All the experiments were performed for three times. In situ hybridization score was analyzed according to *Immunohistochemistry* described.

### CCK-8, EDU, and Ki67 immunofluorescence for cellular proliferation assays

For CCK-8 assay, HNE1 and CNE2 cells were digested and seeded in 96-well plates with 1000 cells per well after 24 h transfected with siHBP1 or NC. After 6 h culture at 37 °C in 5% CO_2_ incubator, cells were incubated with 10 μl CCK-8 (5 mg/ml, Sigma-Aldrich, St. Louis, MO, USA) for 2 h in 37 °C, then the absorbance was measured at 450 nm by Paradigm Dectection Platform (BECK MAN, S. Kraemer Boulevard Brea, CA) and normalized to that of 0 d. We measured the absorbance for 0 d, 1 d, 2 d, 3 d, 4 d, and 5 d. For Ki67 (BBI Life science, Shanghai, China) and EDU (Ribobio, Guangzhou, China) assay, HK1, HNE1, and CNE2 cell lines were transfected with 40 nM siHBP1 and siNC, or 3 μg pre-miR-29c and pSUPER vector for 24 h, respectively, then 20,000 cells for each group were seeded into 6-well plates for Ki67 assay, and 2000 cells for each group were seeded into 96-well plates for EDU assay. We performed the Ki67 and EDU assay according to the manufacturer’s protocol. A total five fields of sample view at 20× were scored for Ki67 or EDU positive cells. The Integrated Optical Density (IOD) and area were measured by Image-Pro Plus version (MediaCybernetics, USA); IOD/area was set as a semi-quantitative index for the expression of Ki67 and EDU-stained cells.

### Immunofluorescence

Cells were transfected with 40 nM siHBP1 and siNC for 24 h and then seeded in 6-well plates as described above. After 24 h culture, cells were fixed with 4% paraformaldehyde solution and followed by the protocols as previously described^41^. The photographs were taken at five different fields at 20× under BX60 fluorescence microscope (Olympus, Japan) and were scored for HBP1 expression. The anti-HBP1 (Millipore, Billerica, MA,USA, 1:200) was a rabbit-IgG antibody and the Integrated Optical Density (IOD) and area were measured by Image-Pro Plus software (MediaCybernetics, USA), IOD/area was set as a semi-quantitative index for the expression of HBP1 protein.

### Transwell assay

HK1, HNE1, and CNE2 cells were transfected with miR-29c mimics or siHBP1 as described above. 5.0 × 10^4^ cells were resuspended in 200 μl 1640 medium without FBS and seeded into matrigel-covered Transwell Chamber (Costar, USA) (upper chamber), meanwhile, 700 μl 1640 medium containing 15% FBS was added to the lower chamber. We terminated the culture when cells were invaded through the membrane and migrate into the lower side of the chamber membrane, then the cells which invaded into the lower side were fixed and dyed with crystal violet solution (0.1%, Sangon Biotech Co, Ltd, Shanghai, China) for 5 min, the cells on the upper side were wiped off using cotton swabs and the lower side of the membrane was washed with appropriate PBS solution and photographs were taken under CKX41 optical microscope (Olympus, Japan). Photographs with five fields of every group were taken and the cells were counted using Image-Pro Plus software.

### Western blotting analysis

HK1, HNE1, and CNE2 cell lines were transfected as described above. After 48 h transfection, proteins were extracted in the defined volume of RIPA lysis buffer containing 1.0 mM PMSF, protease inhibitor cocktail and DTT, and 50 μg proteins was loaded in SDS-PAGE gel electrophoresis^10^. The proteins were transferred to PVDF Membrane (Milipore, Darmstadt, Germany) which were incubated with following primary antibodies: anti-HBP1 (Milipore, Darmstadt, Germany), anti-ZO-1, anti-E-cadherin, anti-N-cadherin, anti-Vimentin, anti-β-catenin, anit-MMP9, anti-NF-κB, anti-caspase 3 and cleaved caspase 3, anti-caspase 7, anti-PARP and cleaved PARP, anti-caspase 9 and cleaved caspase 9, anti-p18^INK4C^ (p18), anti-p21^Waft/Cip1^ (p21), anti-p27^Kip1^ (p27), anti-Cyclin D1, anti-Cyclin D3, anti-CDK2, anti-CDK4, anti-CDK6 (Cell Signaling Technology, Danvers MA, USA), and β-actin (ABclonal, Cambridge, MA, USA) as an internal reference. Then the PVDF membranes were incubated with HRP-linked anti-Rabbit or anti-Mouse IgG antibody according to the isotypes of the primary antibody. The membrane was imaged using ChemiDoc MP System (Bio-Rad, Hercules, CA, USA) and the images were analyzed using Image Lab Software (Bio-Rad, CA, USA).

### Cell apoptosis and cycle assay

Cells were transfected with siHBP1 and siNC as described as above. Cells were digested and washed with PBS solution at 48 h post-transfection. Apoptosis assay were performed using Annexin V-EGFP Apoptosis Detection Kit (BIOBOX, Nanking, China) according to the manufacture’s protocol. For cell cycle assay, cells were resuspended with 300 μl PBS, then 700 μl pre-cooled methanols were added drop wise to the cell suspension and fixed at −20 °C for at least 12 h. Following procedures were performed according to the manufacturer’s instructions by Cell Cycle Staining Kit (Multi Science, Hangzhou, China).

### Real-time quantitative -PCR (RT-qPCR)

Total miRNA and mRNA were isolated from cells with Trizol Reagent (Life Technologies, Carlsbad, CA, USA). Then miRNA and mRNA were extracted using miRNeasy Mini Kit (Qiagen, Hilden, Germany). For miRNA expression analysis, reverse transcription was performed using miScript II RT Kit (Qiagen, Hilden, Germany), and real-time quantitative PCR (RT-qPCR) was conducted by miScript SYBR Green PCR Kit (Qiagen, Hilden, Germany) on CFX96 Real-Time PCR Detection System (Bio-Rad, Hercules, CA, USA), U6 was used as a loading control. For mRNA expression analysis, cDNA was synthesized using Revert Aid First SYBR Green PCR Kit (Thermo Fisher Scientific, Waltham, MA, USA). Then RT-qPCR was used 2 × SYBR Green qPCR Master Mix (Biotool, HOU, USA) by CFX96 Real-Time PCR Detection System (Bio-Rad, Hercules, CA, USA), GAPDH was used as an internal control for mRNA. The expression level of miRNA or mRNA was measured using Bio-Rad Manager Software. All primers sequences were listed in Table S4.

### Chromatin immunoprecipitation (ChIP) assay

ChIP assay was carried out as previously described^31^. HK1 Vector and HK1 shHBP1 cells (about 1.0 × 10^7^) were fixed by 1% paraformaldehyde (PFA; Biosharp, Hefei, China) for protecting the cross-linking state of protein and DNA at 37 °C, then were neutralized with 0.125 M (final concentration) glycine (Biosharp, Hefei, China). Cells were collected and disrupted by 600 μl SDS lysis buffer (1% SDS, 10 mM EDTA, 50 mM Tris HCl, PH = 8.0) for 20 min on ice. Then the lysate was sonicated (Cole-Parmer Instruments CP 130; Vernon Hills, Illinois, USA) for 8 cycles of 20 s sonication with an interval of 30 s, and centrifuged at 13,000 r.p.m. (Centrifuge 5424R; Eppendorf, Germa) for 15 min at 4 °C. 20% of cell supernatant as Input, divided the reminding into two samples which were incubated with 4 μg normal mouse anti-IgG (Santa Cruz Biotechnology, CA, USA) or mouse monoclonal anti-HBP1 (Santa Cruz Biotechnology, CA, USA), respectively, and rotated overnight at 4 °C. The Protein A/G immunoprecipitation magnetic beads (Bimake, Shanghai, China) were washed with wash buffer (50 mM Tris, 150 mM NaCl, 0.3% Triton 100, pH 7.5) for twice, and then incubated with above samples for 2 h at 4 °C, respectively. Two samples were washed for at least 3 times and then placed on the magnet holder for 2 min, removed the supernatant and added 100 μl 10% Chelex-100 (Bio-Rad, Hercules, CA, USA). The mixed solutions were incubated at 99 °C for 10 min and were then centrifuged at 15,000 r.p.m. for 5 min at 4 °C. DNA was purified by Gel Extraction Kit (OMEGA, Norcross, GA, USA) and its levels were detected by RT-qPCR. HBP1 binds preferentially to the sequence 5′-TTCATTCATTCA-3′ of targets promoter^42,43^, and the promoter primers were designed via combining with the high affinity-sites of HBP1, also see Table S4. ChIP assay for HK1 Vector and HK1 shHBP1 cells were performed at least three times.

### In vivo assays

In miR-29c agomir treatment tumor model, BALB/c nude mice (female, 4–5 weeks old and 16–18 g) were randomly divided into two groups (*n* = 6 each) and injected subcutaneously, with 100 μl 1640 medium containing 1.0 × 10^6^ HK1 cells into the flank of either axillary. After the length and width of tumors grew about 5 mm × 5 mm (about 10 d), 2 nmol (volume, 50 μl) of 2′-O-Methyl-conjugated 5′-Cholesterol (2′Ome-5′Chol) modified miR-29c or NC agomir (RiboBio Co., Ltd, Guangzhou, China) was injected into intratumorally twice a week, a total of 3 weeks (12 nmol/each mouse). Meanwhile, the length (*L*) and width (*W*) (mm) of tumors were measured with micrometer twice a week. Mice were sacrificed 31 days after initial injection and tumors were stripped from skin tissue. In siHBP1 treatment tumor model, 2.0 × 10^6^ HK1 cells were subcutaneously injected into the flank of either axillary of BALB/c nude mice (female, 4–5 weeks old and 16–18 g, *n* = 5). After 5 days since initial xenografts, 2 nmol (volume, 40 μl) 2′Ome-5′Chol siHBP1 or NC (RiboBio Co., Ltd, Guangzhou, China) was injected into intratumorally twice a week, a total of 3 weeks (12 nmol/each mouse). Mice were sacrificed 23 days after initial injection and tumors were stripped from skin tissue. Tumors volume (*V*: mm^3^) was calculated with the formula *V* = 1/2 × *L* × *W*^2^. All tumor tissues were fixed in 4% paraformaldehyde solution for 36 h, then embedded by paraffin and sliced up. H&E staining, in situ hybridization and immunohistochemistry were performed following with above steps.

### Statistical analyses

All results are presented as Mean ± SD. Student’ *t*-test was used to analysis two-group data and one-way ANOVA was used for three-group data. Kaplan-Meier analysis was applied to survival analysis in patients with NPC. All graphs and statistical were performed by employing GraphPad Prism 5.0 software (GraphPad Software Inc). All graphs were summarized from three independent experiments. *P* value less than 0.05 (*p* < 0.05) was regarded as statistically significant.

## Electronic supplementary material

supplementary material

Supplemental Figure 1

Supplemental Figure 2

Supplemental Figure 3

Supplemental Figure 4

Supplemental Table 1

Supplemental Table 2

Supplemental Table 3

Supplemental Table 4
