# Supplementary material for: HMG-box transcription factor 1: a positive regulator of the G1/S transition through the Cyclin-CDK-CDKI molecular network in nasopharyngeal carcinoma
Source: Cell Death Dis. 2018 Jan 24;9(2):100. doi: 10.1038/s41419-017-0175-4 (PMC5833394; doi:10.1038/s41419-017-0175-4)
Supplement: Supplementary file 1 — supplementary material [file 41419_2017_175_MOESM1_ESM.docx]

**Supplementary Meterial**

**Figure S1 Prediction of binding sites between hsa-miR-29c and HBP1.** (A) MiR-29c down-regulated HBP1 mRNA expression after transfection with pre-miR-29c. Coll1a was used as a positive control. (B) Predicted alignment of hsa-miR-29c and HBP1 by miRanda system. (C) Conserved sites for miRNA families broadly conserved among HBP1 mRNA sequences by TargetScan software. (D) Predicted consensus sequence of HBP1 and hsa-miR-29c by TargetScan. (E) HBP1 full-length cDNA were cloned from NP69, HNE1, HK1 and CNE2 cell lines by agarose gel electrophoresis. (F) Dysregulated expression of HBP1 among NPC cells and non-NPC cells. NP69, human nasopharyngeal epithelial cell line; HK1, HNE1 and CNE2, NPC cells. QSG7701, human hepatocyte line; QYG7703, SMMC7721 and HepG2, liver cancer cells. MCF-10A, human mammary epithelial cell line; MCF-7, T47D and MDA-MB-231, breast cancer cells. IOSE-80, human ovarian epithelial cell line; A2780, PA-1 and SKOV3, ovarian cancer cells.

**Figure S2 HBP1 expression levels are decreased by miR-29c in NPC cell lines. (A-C)** [Immunofluorescence](javascript:void(0);) and *in situ* hybrization for detecting HBP1 and miR-29c expression levels in HK1, HNE1 and CNE2 cells after transfection with empty vector or pre-miR-29c, siNC or siHBP1. Semi-quantitative analysis for HBP1 expression in NPC cell lines after transfection with pre-miR-29c or siHBP1. IOD/area was normalized to vector or siNC. Scale bars, 50 μm.

**Figure S3 Ensemble and** **GO annotations of transcription factor HBP1.** (A) HBP1 locates at 7q22.3 (Chromosome 7: 107 168 290-107 203 200). (B) Detail gene legend of HBP1. (C) GO annotation for the process, functions and component of HBP1 from GO term.

**Figure S4 2’Ome-5’Chol-modified miR-29c agomir and siHBP1 decreased HBP1 expression *in vitro*.** (A) RT-qPCR analysis for miR-29c miRNA levels in HK1 cells after treatment with NC or miR-29c agomir. (B) RT-qPCR analysis for HBP1 mRNA levels in HK1 cells after treatment with NC or miR-29c agomir and siNC or siHBP1. (C) Western blotting analysis for HBP1 protein as above (B).

**Table S1** **Dysregulated microRNAs filtrated from NPC GEP data (GSE12452) by SAM analysis.**

**Table S2 Differential expression levels of MiR-29c and HBP1 between NPE tissues and NPC tissues (Data from GSE12452).**

**Table S3** **Has-miR-29c** **target-genes (Data from TargetScan).**

**Table S4 Primers, DNA Oligonucleotides and RNA sequences.**
