# Supplementary figures and images for: HMG-box transcription factor 1: a positive regulator of the G1/S transition through the Cyclin-CDK-CDKI molecular network in nasopharyngeal carcinoma
Source: Cell Death Dis. 2018 Jan 24;9(2):100. doi: 10.1038/s41419-017-0175-4 (PMC5833394; doi:10.1038/s41419-017-0175-4)

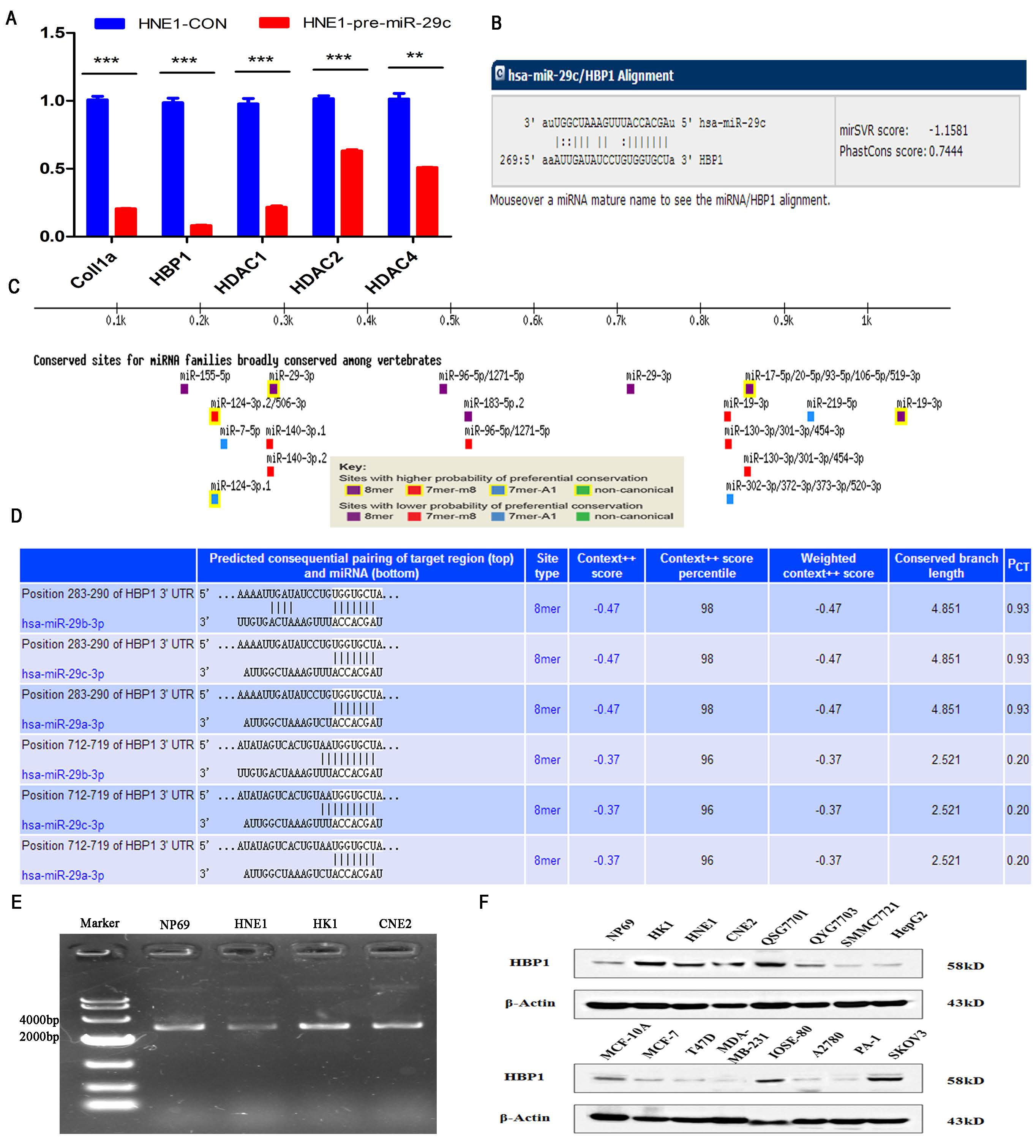

Supplement: Supplementary file 2 — Supplemental Figure 1 [file 41419_2017_175_MOESM2_ESM.tif]

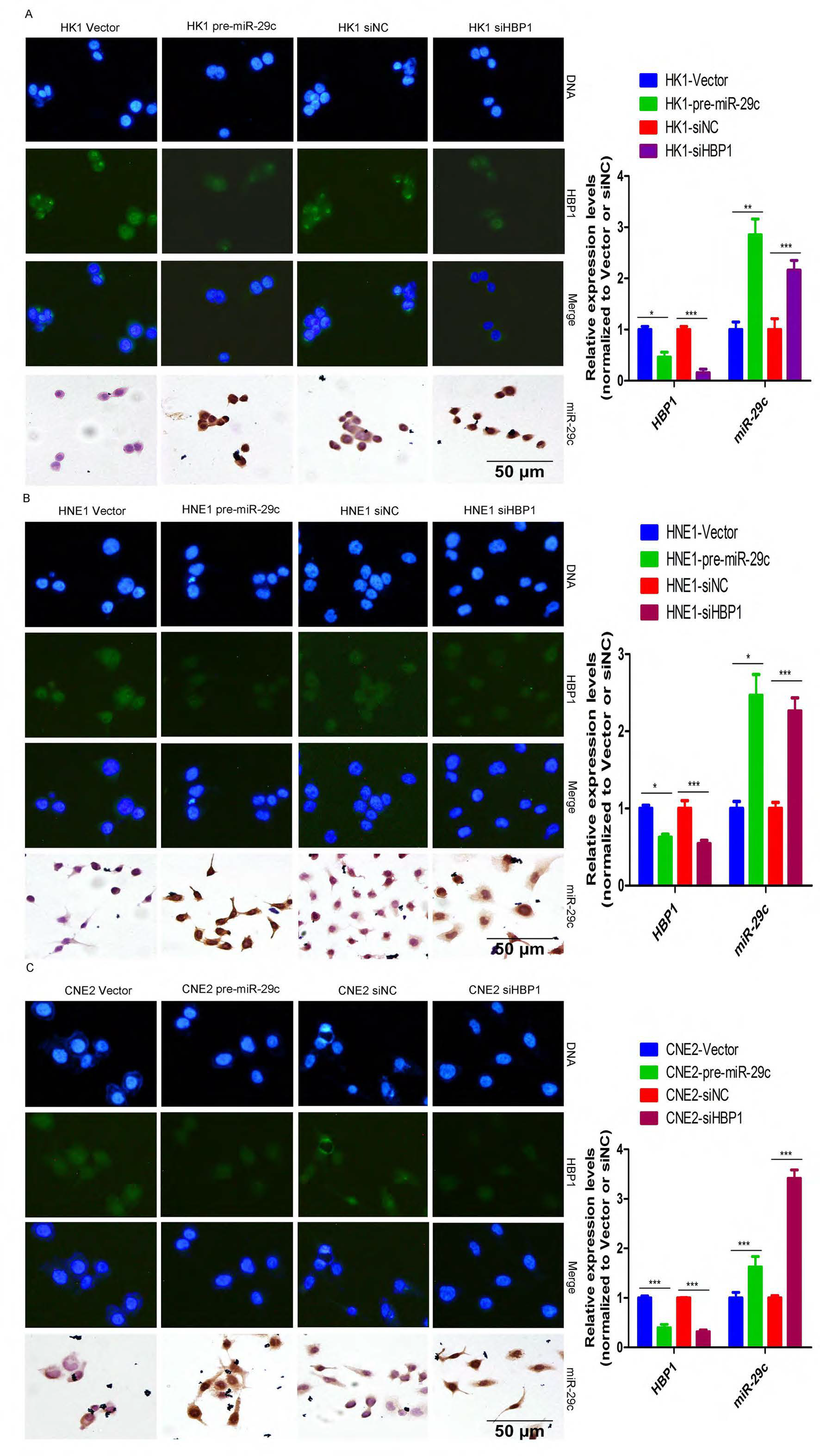

Supplement: Supplementary file 3 — Supplemental Figure 2 [file 41419_2017_175_MOESM3_ESM.tif]

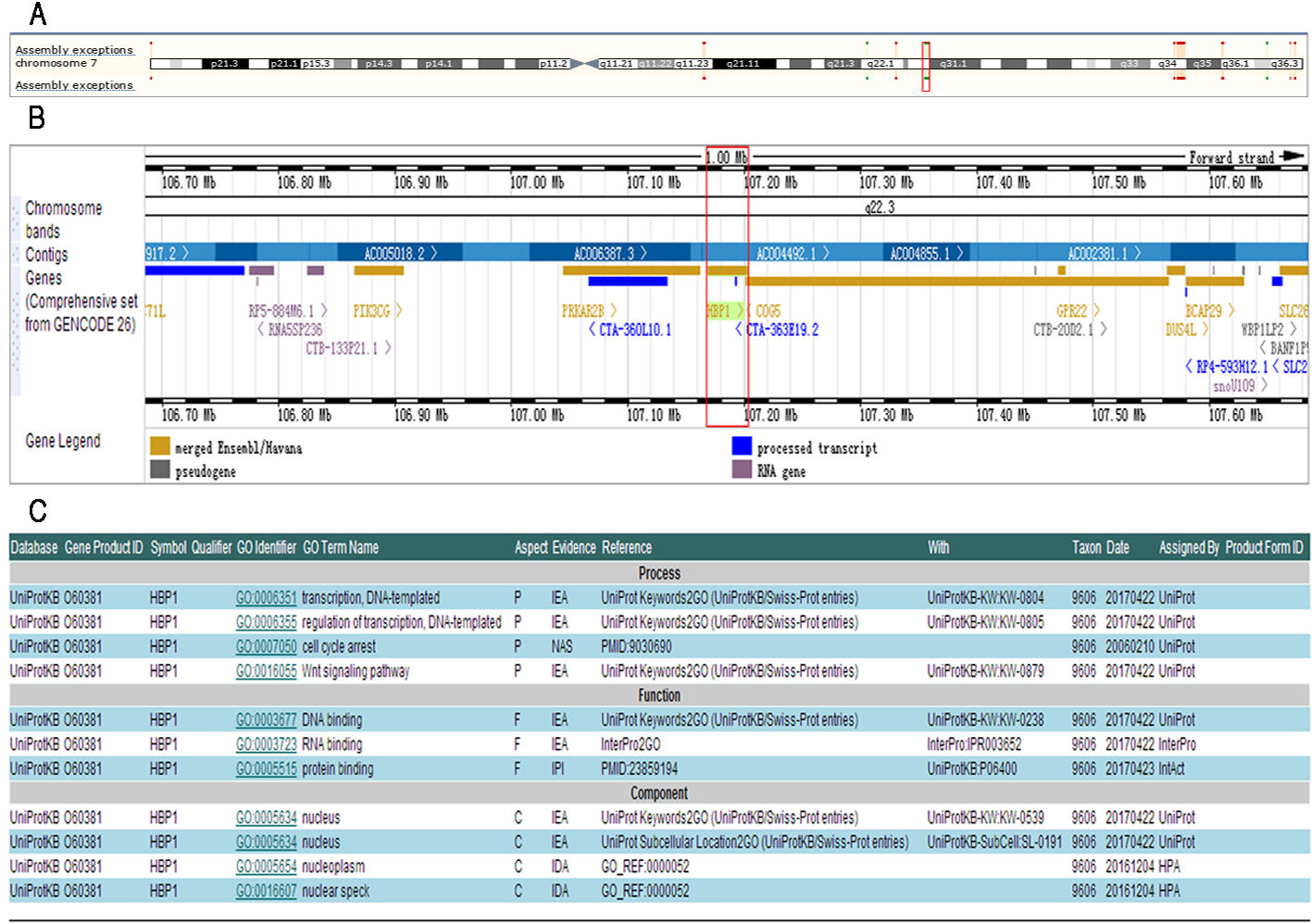

Supplement: Supplementary file 4 — Supplemental Figure 3 [file 41419_2017_175_MOESM4_ESM.tif]

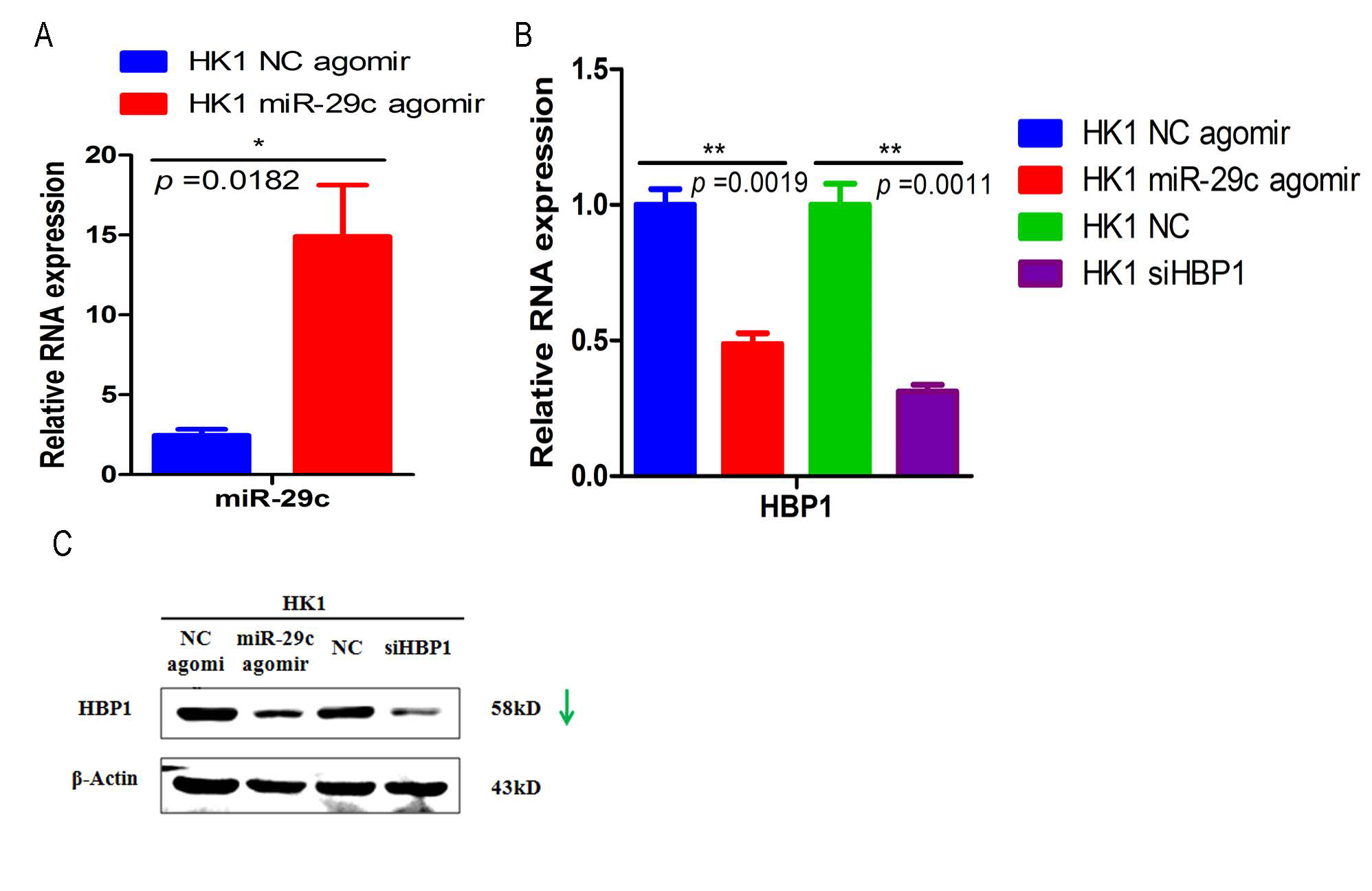

Supplement: Supplementary file 5 — Supplemental Figure 4 [file 41419_2017_175_MOESM5_ESM.tif]
