# Supplementary material for: HMG-box transcription factor 1: a positive regulator of the G1/S transition through the Cyclin-CDK-CDKI molecular network in nasopharyngeal carcinoma
Source: Cell Death Dis. 2018 Jan 24;9(2):100. doi: 10.1038/s41419-017-0175-4 (PMC5833394; doi:10.1038/s41419-017-0175-4)
Supplement: Supplementary file 9 — Supplemental Table 4 [file 41419_2017_175_MOESM9_ESM.doc]

**Table S4. Primers, DNA Oligonucleotides and RNA sequences.**

| **Name** | **Forwards Primer (5'-3')** | **Reverse Primer (5'-3')** |
| --- | --- | --- |
| **Primers for HBP1 3’ UTR wild type and mutant site** | | |
| HBP1 3’ UTR wild type | CGCGTGCTTGAAAATTGATATCCTGTGGTGCTAAAGTACAGTAGAAAGAGAGGA | AGCTTCCTCTCTTTCTACTGTACTTTAGCACCACAGGATATCAAT TTTCAAGCA |
| HBP1 3’ UTR mutant | CGCGTGCTTGAAAATTGATATCCTGTGGCCCTAAAGTACAGTAGAAAGAGAGGA | AGCTTCCTCTCTTTCTACTGTACTTTAGGGCCACAGGATATCAAT TTTCAAGCA |
| **Primers for pre-miR-29c gene** | | |
| pre-miR-29c | GATCTATCTCTTACACAGGCTGACCGATTTCTCCTGGTGTTCAGAGTCTGTTTTTGTCTAGCACCATTTGAAATCGGTTATGATG TAGGGGGAA | AGCTTTCCCCCTACATCATAACCGATTTCAAATGGTGCTAGACAAAAACAGACTCTGAACACCAGGAGAAATCGGTCAGCCTGTGTAAGAGATA |
| **Primers for shHBP1 consturct** | | |
| shHBP1 | GATCTTACCTCAGACATACCAGAATTCAAGAGATTCTGGTATGTCTGAGGTA TTTTTA | AGCTTAAAAATACCTCAGACATACCAGAATCTCTTGAA TTCTGGTATGTCTGAGGTA A |
| **Primers for q-PCR** | | |
| Coll1a | AGCCAGCAGATCGAGAACAT | TCTTGTCCTTGGGGTTCTTG |
| HDAC1 | GGAAATCTATCGCCCTCACA | AACAGGCCATCGAATACTGG |
| HDAC2 | CACTGCCGAAGAAATGACAA | TCCTCCAGCCCAATTAACAG |
| HDAC4 | TCCGTTCCATGGAGAAAGTC | CTTCGAGGGAGTGCTACAGG |
| HBP1 | CCGTGAAAATGAGGTGGACT | GAAGGCTGGTTCACTCTTCG |
| CDK1 | TTTCTTTCGCGCTCTAGCCA | GGTAGATCCGCGCTAAAGGG |
| CDK2 | TGGCGCTTCATGGAGAACTT | ACATCCAGCAGCGTGTCC |
| CDK4 | TTGGCAGCTGGTCACATGGT | TCAGATCAAGGGAGACCCTCAC |
| CDK6 | AGTCTGATTACCTGCTCCGC | TCCAGAATCATTGCACCTGAG |
| CCNB1 | CGCCTGAGCCTATTTTGGTTG | AGTGACTTCCCGACCCAGTA |
| CCND1 | TGTGCCACAGATGTGAAGTT | CTTGGGGTCCATGTTCTGCT |
| CCND3 | AAACTTGGCTGAGCAGAGCA | GCTCCTCACATACCTCCTCG |
| CCNE1 | CCCATCATGCCGAGGGAG | TATTGTCCCAAGGCTGGCTC |
| P16 | TCATCATGACCTGGATCGGC | CTTCGGCTGACTGGCTGG |
| P21 | AGTCAGTTCCTTGTGGAGCC | GACATGGCGCCTCCTCTG |
| P27 | TAATTGGGGCTCCGGCTAAC | GAAGAATCGTCGGTTGCAGGT |
| P53 | TGCTCAAGACTGGCGCTAAA | CAGTCTGGCTGCCAATCCA |
| β-Actin | TCACCAACTGGGACGACATG | GTCACCGGAGTCCATCACGAT |
| GAPDH | AACGGATTTGGTCGTATTGG | TTGATTTTGGAGGGATCTCG |
|  | | |
| **Sequence for whole HBP1 cDNA** | | |
| HBP1 | ATATAACCCCCAGGGGGTTGAGGGGA | GCGCCAGAGATAAGCAACTTTTTTTTCC |
| ***In situ* hybridization** [**probe**](javascript:void(0);) **for miR-29c** | | |
| miR-29c | TAGCACCATTTGAAATCGGTTA |  |
| **Sequence for siHBP1 or siNC** | | |
| siHBP1 | UACCUCAGACAUACCAGAATT | UUCUGGUAUGUCUGAGGUATT |
| siNC | UUCUCCGAACGUGUCACGUTT | ACGUGACACGUUCGGAGAATT |
| **Sequence for ChIP primers** | | |
| prCCND1-1* | GCCCCATAAATCATCCAGGC | ATAGCCAAGCCTCAGAGCAT |
|  |  |  |
|  |  |  |
| prCCND1-2 | TATTTTTTGAGCGAGCGCATG | ACTAATTTAGCATGCAAGGACGG |
| prCCND3 | CGCATTCCTTAGAGCAAGCA | GGACTCTAGTCACCCAGGAA |
| prCDK2 | CGTTCATCTCTTTCCTCCTCT | GAGATTAGGAAAAGGGGTCTGA |
| prCDK4-1 | GACAGTAATGTTCCCATGAAGT | CAGAAGACGTCCTCTCTGTCT |
| prCDK4-2 | CAAATGAGTAGTCACAAGGTTTG | ATGTGCAAGAAAGTTACCACCA |
| prCDK4-3 | ACCAAAAACAAAATAAAAACCCA | AACATACTCTCATTTGTGTCA |
| prCDK6 | GTGGTAGAAAGAATGTGTTT | CATGAGCCACCGTGCCCG |
| prP16 | CCTTCCAATGACTCCCTC | AACCTTCCTAACTGCCAAA |
| prP21 | TTCTTACTTCGTTTCAGTCAAGA | TATATAAATCTTTGTGTAAAATG |
| prP53 | TCTCATTCTCCAGGCTTCAGA | TTATGAGGATATAGAATTTTTCT |
| prGAPDH | tcttgactcaccctgccct | acaaaggcactcctggaaac |

Abbreviations: HDAC, histone deacetylase; CDK, Cyclin-dependent kinase; CCNB1, cyclin B1; CCND1, cyclin D1; CCND3, cyclin D3; P21, cyclin dependent kinase inhibitor 1A/P21CIP1; P27, cyclin dependent kinase inhibitor 1B/P27KIP1.

* pr was represented as promoter of targeting genes. Severial gene promoters and HBP1 has two or more binding sites.
